# Dysfunctional endocannabinoid CB1 receptor expression and signaling contribute to skeletal muscle cell toxicity induced by simvastatin

**DOI:** 10.1038/s41419-023-06080-9

**Published:** 2023-08-23

**Authors:** Hilal Kalkan, Elisabetta Panza, Ester Pagano, Giuseppe Ercolano, Claudia Moriello, Fabiana Piscitelli, Mónika Sztretye, Raffaele Capasso, Vincenzo Di Marzo, Fabio Arturo Iannotti

**Affiliations:** 1 Endocannabinoid Research Group, Institute of Biomolecular Chemistry (ICB), National Research Council (CNR), Pozzuoli, NA 80078 Italy; 2 Department of Pharmacy, University Federico II of Naples Italy, Naples, Italy; 3 Department of Physiology, Faculty of Medicine, University of Debrecen, 4032 Debrecen, Hungary; 4 Department of Agricultural Sciences, University of Naples Federico II, Via Università 100, 80055 Portici, Italy; 5 Institut Universitaire de Cardiologie et de Pneumologie de Québec and Institut Sur la Nutrition et Les Aliments Fonctionnels, Centre NUTRISS, Université Laval, Quebec City, QC G1V 0A6 Canada

**Keywords:** Mechanisms of disease, Apoptosis

## Abstract

Statins are the most prescribed lipid-lowering agents worldwide. Their use is generally safe, although muscular toxicity occurs in about 1 in 10.000 patients. In this study, we explored the role of the endocannabinoid system (ECS) during muscle toxicity induced by simvastatin. In murine C2C12 myoblasts exposed to simvastatin, levels of the endocannabinoids AEA and 2-AG as well the expression of specific miRNAs (in particular miR-152) targeting the endocannabinoid CB1 gene were increased in a time-dependent manner. Rimonabant, a selective CB1 antagonist, exacerbated simvastatin-induced toxicity in myoblasts, while only a weak opposite effect was observed with ACEA and GAT211, selective orthosteric and allosteric agonists of CB1 receptor, respectively. In antagomiR152-transfected myoblasts, simvastatin toxicity was in part prevented together with the functional rescue of CB1. Further analyses revealed that simvastatin in C2C12 cells also suppresses PKC and ERK signaling pathways, which are instead activated downstream of CB1 receptor stimulation, thus adding more insight into the mechanism causing CB1 functional inactivation. Importantly, simvastatin induced similar alterations in skeletal muscles of C57BL/6 J mice and primary human myoblasts. In sum, we identified the dysregulated expression of the endocannabinoid CB1 receptor as well as the impairment of its downstream signaling pathways as a novel pathological mechanism involved in statin-induced myopathy.

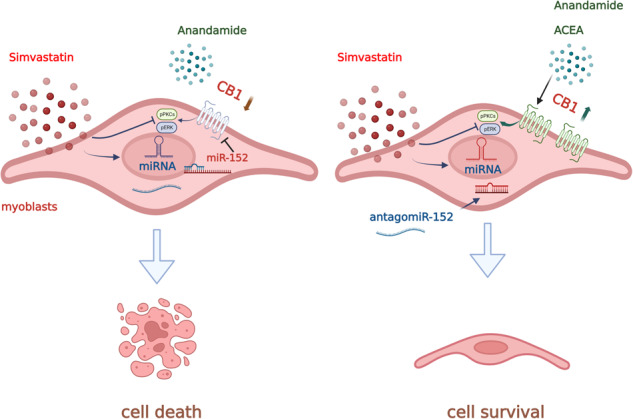

## Introduction

Statins are a class of drugs widely used worldwide to reduce cholesterol levels in the blood. In pathological conditions such as hypercholesterolemia, the excessive amount of low-density lipoprotein (LDL) also called “bad” cholesterol, leads to fat deposits in the artery and vessel walls, with consequent formation of atheromatous plaques, which, when becoming unstable, cause atherosclerotic cardiovascular diseases (ASCVDs). According to estimates of the Global Burden of Disease Study 2019, ASCVDs still are the first leading cause of death globally [1, 2]. Statins are the most effective drugs for reducing LDL cholesterol and reducing cardiovascular event risk in both primary and secondary prevention [3]. As lipid-lowering agents, statins exert beneficial effects through the selective and competitive inhibition of hydroxymethylglutaryl-CoA (HMG-CoA) reductase, the enzyme responsible for converting HMG-CoA to mevalonate in the cholesterol synthesis pathway in both hepatic and non-hepatic cells [4]. Even though this mode of action is common to all statins, they may differ in terms of pharmacokinetics, pharmacodynamics, bioavailability and safety properties [5]. Cross-sectional and observational studies revealed that the most frequently used statin is atorvastatin (42.8%), followed by simvastatin (27.6%) and rosuvastatin (22.8%) [6, 7]. All statins are generally well-tolerated. However, their use may cause serious side effects, especially in patients with multimorbidity and/or taking several different medications. The major reason for statin therapy discontinuation is because of skeletal muscle toxicity, which can occur in 10–30% of patients and includes myalgia, myopathy and myositis with elevated CK (creatinine kinase) and, in more severe cases (fortunately, less than 1%) rhabdomyolysis [8, 9]. Lifestyle interventions, vitamins supplementation and coenzyme Q10 were found to be ineffective at reducing the risk, or improving symptoms of statin-associated myopathy [9, 10].

The endocannabinoid system (ECS) is a complex modulatory system involved in the fine-tuning of cell responses to various intrinsic as well as extrinsic stimulants through a complex cascade of receptor activation, gene expression, substrate mobilization, and enzyme reactions. In particular, the synthesis on demand of the two lipid mediators, anandamide (AEA) and 2-arachidonoylglycerol (2-AG), followed by the activation of two specific metabotropic receptors named cannabinoid receptor of type 1 (CB1) and type 2 (CB2), as well as of the transient receptor potential vanilloid 1 (TRPV1) channel, is the foremost mechanism through which the ECS regulates a variety of physiological and pathophysiological processes in our body.

Despite the acknowledged importance of the ECS and the potential use of cannabinoid-based drugs to treat numerous human disorders [11–14], to date, little is known about the role and implication of this system in skeletal muscle development and disease. In the last few years, we demonstrated that activation of CB1 by 2-AG is a major mechanism acting to promote the proliferation and self-renewal of satellite and myoblast cells and halt their lineage commitment to mature myotubes. Additionally, we found that excessive production of endocannabinoids leading to the dysregulation of CB1 receptor and associated signaling contributes to the irreversible degeneration of skeletal muscle tissues caused by Duchenne muscular dystrophy, the most frequent and detrimental form of hereditary myopathy [15, 16]. Other studies show that excessive activity of CB1 in rat and human muscle cells negatively modulates glucose and fatty acid metabolism [17, 18]. There is also evidence that CB1 receptors are localized on mitochondrial membranes in striated muscles, where their activation positively regulates Krebs’s cycle activity [19]. In summary, the activity and involvement of the ECS in skeletal muscle disorders is a topic of great interest due to its possible future therapeutic implications.

Based on this background knowledge, we have endeavored to understand the role played by the ECS in simvastatin-induced myotoxicity in both murine C2C12 and skeletal muscle tissues of C57BL/6 mice using a multi-technical approach based on molecular biology, biochemical and pharmacological analyses. Importantly, the role of ECS in simvastatin-induced cell toxicity was also explored in human primary myoblasts.

## Results

### Simvastatin causes alterations of ECS activity in C2C12 cells

**Fig. 1:**
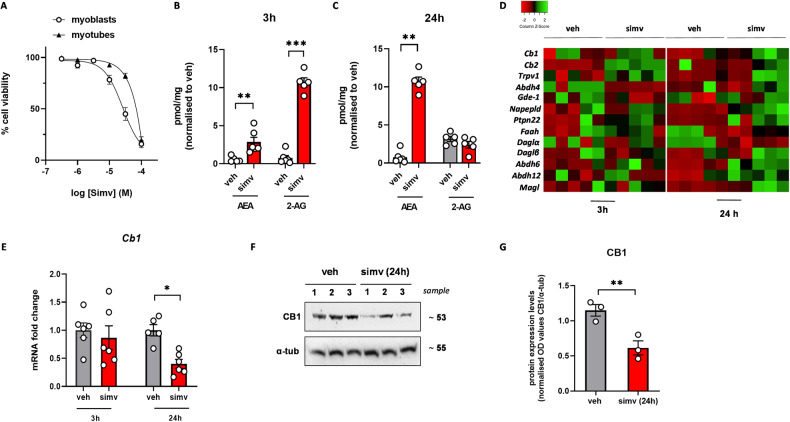
Effect of simvastatin on cell viability and ECS activity in C2C12 cells. **A** concentration-response curves obtained measuring cell viability in C2C12 myoblasts and myotubes exposed to simvastatin for 24 h by MTT assay. Levels of AEA and 2-AG measured in C2C12 myoblasts exposed to simvastatin (30 µM) for 3 (**B**) and 24 (**C**) h. **D** Heatmap showing the expression of ECS-related genes in myoblasts exposed to simvastatin for 3 and 24 h. Green, upregulated; Red, downregulated. **E** Bar graph with individual points showing mRNA expression levels of *Cnr1* in myoblasts treated with vehicle (DMSO) or simvastatin for 3 and 24 h. Representative blot (**F**) and bar graph with individual points **(G)** showing the expression of CB1 protein in myoblasts treated with simvastatin for 24 h. Each bar is the mean ± S.E.M. from 3 to 5 independent biological samples. * = *p* ≤ 0.05; ** = *p* ≤ 0.01; *** = *p* ≤ 0.001 versus the indicated experimental groups.

To explore whether in skeletal muscle cells statin exposure could induce changes in ECS activity, we used C2C12 cells, a murine myoblast cell line commonly used for their ability to rapidly exit the cell cycle upon serum withdrawal and form differentiated myotubes [20]. In good agreement with previous studies [21], we found that myoblasts were more sensitive to simvastatin than myotubes (Fig. 1A). In myoblasts exposed to simvastatin 30 μM (corresponding to the EC_50_ value, Fig. 1A), we found that the levels of both AEA and 2-AG were significantly increased compared to control (vehicle-treated) cells (Fig. 1B) only after 3 h of exposure to simvastatin. On the other hand, after 24 h, AEA levels were further increased (~10 folds), unlike those of 2-AG that resulted no longer higher than those detected in control cells (Fig. 1C). Using quantitative PCR (qPCR), we found that changes in AEA and 2-AG levels corresponded to changes in the expression of genes encoding enzymes deputed to their synthesis (*Abh4, Gde-1, Napepld* for AEA and *Dagla*, and *Daglb* for 2-AG) and catabolism (*Faah* for AEA and *Abdh6*, *Abdh12* and *Magl* for 2-AG). Furthermore, we found that simvastatin, in a time-dependent manner, significantly reduced mRNA expression levels of *Cnr1* (Cannabinoid Receptor 1), *Cnr2* (Cannabinoid Receptor 2) and *Trpv1* (Transient receptor potential vanilloid 1) genes (Fig. 1D). The bar graph in Fig. 1E shows the quantification of *Cnr1* mRNA expression in myoblasts exposed to simvastatin 30 μM for 3 and 24 h. Changes in *Cnr1* expression were then confirmed by western blot analysis (Fig. 1F and G). In myotubes exposed to simvastatin, AEA and 2-AG levels were unchanged and the expression of *Cnr1* only tended to be reduced (Supplementary Fig. 1). Based on these results, we conducted all subsequent experiments in myoblasts using simvastatin 30 μM (Fig. 1A).

### Pharmacological blockade of CB1 receptors worsens simvastatin toxicity in C2C12 cells

**Fig. 2:**
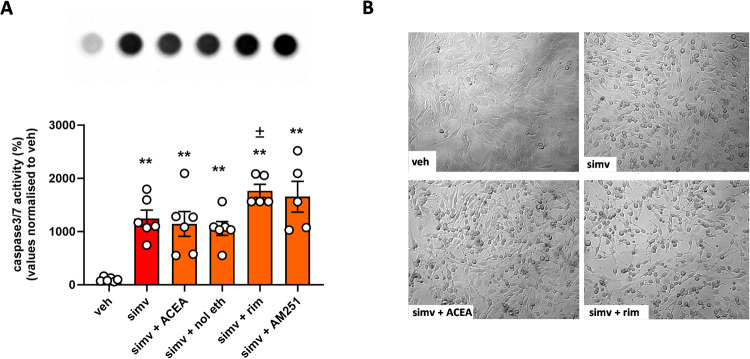
Caspase 3/7 activity in C2C12 myoblasts exposed to simvastatin with or without CB1 drugs. **A** Measurement caspase 3/7 activation in myoblasts exposed to simvastatin (30 µM) for 24 h in the presence or absence of selective CB1 agonists (ACEA 1 µM or noladin ether 1 µM) or antagonists (rimonabant 1 µM or AM251 1 µM). Each bar is the mean ± S.E.M. from 5 to 6 independent biological samples. ** = *p* ≤ 0.01 versus the veh group; ± = *p* ≤ 0.05 versus the group treated with simvastatin alone **(B)** Representative images captured using bright-field light microscopy (10x magnification) showing the effect of simvastatin (24 h) in myoblasts in the presence of ACEA or rimonabant.

To understand whether changes in endocannabinoid levels and CB1 expression were associated with statin-induced skeletal muscle cell toxicity, we exposed C2C12 myoblasts to simvastatin (30 μM) in the presence or absence of selective CB1 receptor full agonists (ACEA and noladin ether) or antagonists (rimonabant and AM251) for 3 and 24 h. Cells viability measured using the MTT assay revealed that the toxic effect of simvastatin 30 μM was not modified by ACEA (1 μM) or noladin ether (1 μM), whereas in the presence of rimonabant (1 μM) and AM251 (1 μM), cell toxicity tended to be worsened at 24 h (Supplementary Fig. 2A). However, measurement of caspase 3/7 activity in the same experimental conditions revealed that C2C12 myoblasts were more susceptible to simvastatin when CB1 receptors were blocked in the presence of rimonabant or AM251, while, also in this case, ACEA or noladin ether did not show significant effects (Fig. 2A). Representative images of cell populations exposed to simvastatin in the presence or absence of ACEA or rimonabant are shown in Fig. 2C.

**Fig. 3:**
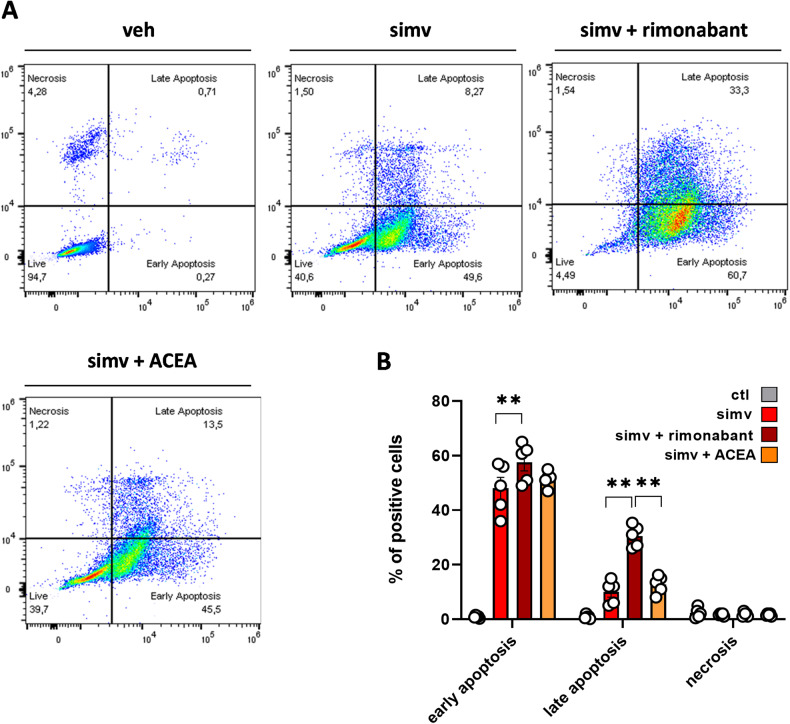
Cell apoptosis measured in C2C12 myoblasts exposed to simvastatin in the presence of rimonabant or ACEA. **A** Representative images of FACS analysis performed in C2C12 myoblasts exposed to simvastatin (30 µM) in the presence or absence of rimonabant (1 µM) or ACEA (1 µM) for 24 h **(B)** Bar graph with individual points showing quantification of results. Each bar is the mean ± S.E.M. from five independent biological samples. ** = *p* ≤ 0.01 versus the indicated experimental groups.

Next, using FACS analysis, we explored more in-depth the role of CB1 receptors during simvastatin-induced muscle cell toxicity. In these experiments, C2C12 myoblasts were exposed to simvastatin (30 µM) in the presence or absence of rimonabant (1 μM) or ACEA (1 μM) for 24 h and subsequently stained with FITC annexin V and propidium iodide (PI) to discriminate early *vs* late apoptosis as well as apoptotic *vs* necrotic cells [22]. The results indicated that in control C2C12 cells exposed to vehicle (DMSO < 0.03%), the number of apoptotic (early or late) and necrotic cells detected ranged between ~0.8% and ~4.5%. The little fraction of dead cells was attributed to the mechanical detachment from Petri dishes as reported also by others [22]. In agreement with the results described above, simvastatin significantly increased the number of cells in both early (~50%) and late (~10%) apoptosis, while no effect of simvastatin on necrosis was detected (Fig. 3A). Interestingly, in myoblasts treated with simvastatin in the presence of rimonabant, the percentage of cells in early and late apoptosis significantly increased (~60% and ~30%, respectively). By contrast, the combination of simvastatin with ACEA did not change the number of apoptotic cells *vs* the group of cells treated with simvastatin alone (Fig. 3A). Quantification of these results is shown in Fig. 3B. Finally, the effect of enhancing CB1 receptor activity induced by endogenous orthosteric agonizts was explored using GAT211, a positive allosteric modulator (PAM) of this receptor. We found that GAT211 had a slight, albeit statistically significant, effect at preventing statin toxicity in myoblasts (Supplementary Fig. 2B).

### Simvastatin causes a miRNA-mediated suppression of CB1 gene expression in C2C12 myoblasts

**Fig. 4:**
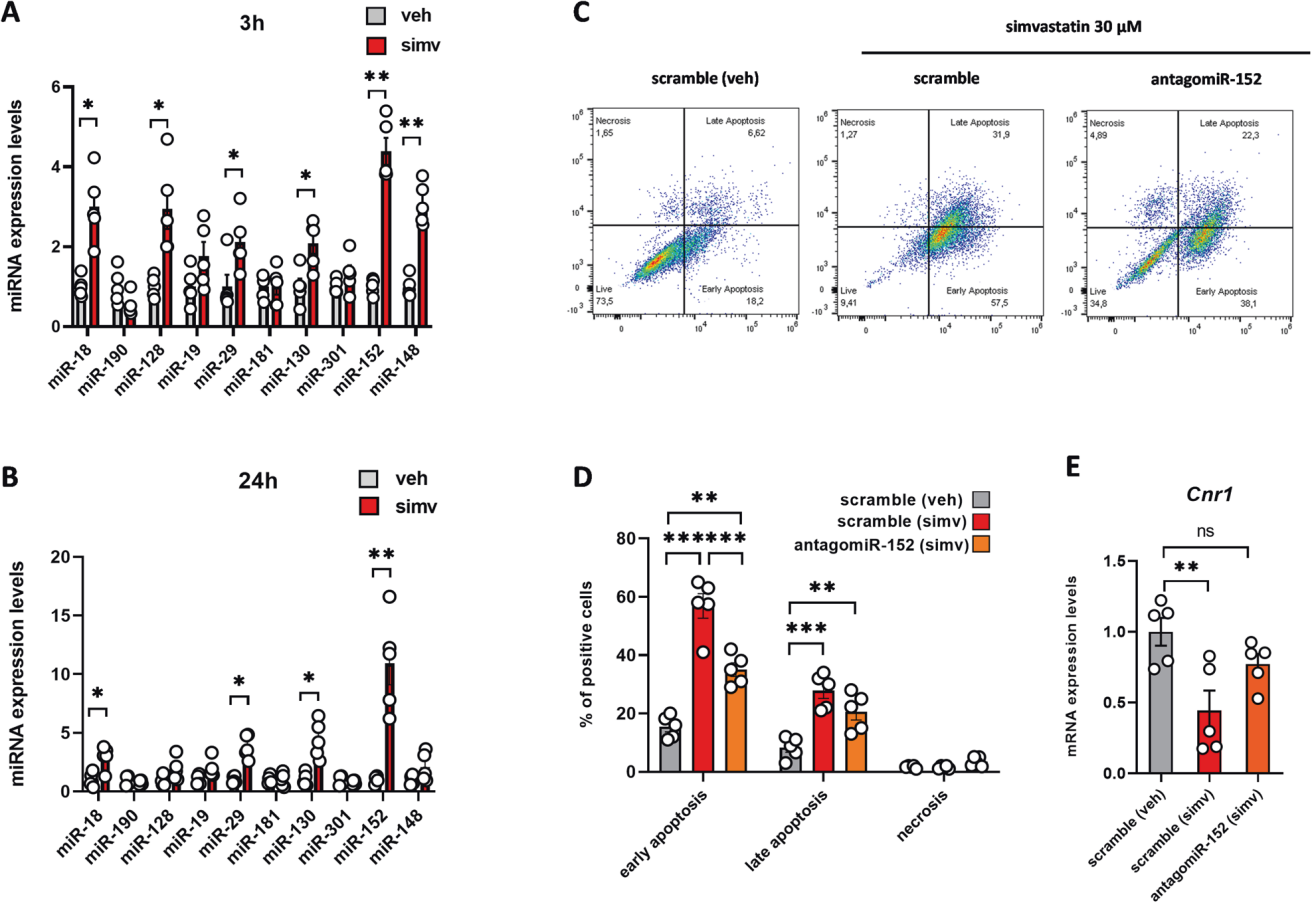
Effect of simvastatin on the expression of miRNAs targeting the *Cnr1* gene in C2C12 myoblasts and cell apoptosis in antagomiR-152 transfected cells. Bar graph with individual points showing the expression of indicated miRNAs in C2C12 myoblasts exposed to simvastatin for 3 **(A)** and 24 **(B)** h. Each bar is the mean ± S.E.M. from five independent biological samples. * = *p* ≤ 0.05; **= *p* ≤ 0.01 versus the indicated experimental group. **C** Representative images of FACS analysis performed in C2C12 myoblasts transfected with control (scramble) or antagomiR-152 sequences and then exposed vehicle (DMSO) or simvastatin (30 µM) for 24 h. **D** Bar graph with individual points showing quantification of simvastatin effects on apoptosis and **(E)** *Cnr1* mRNA expression. Each bar is the mean ± S.E.M. from five independent biological samples. * = *p* ≤ 0.05; ** = *p* ≤ 0.01; *** = *p* ≤ 0.01 versus the indicated experimental groups.

Next, we went on to elucidate the molecular mechanisms through which simvastatin causes time-dependent down-regulation of *Cnr1* (Cb1) gene expression in C2C12 myoblasts. Toward this goal, using bioinformatics tools, we identified a large set of microRNA sequences (miRNAs) targeting the three prime untranslated region (3′-UTR) of *Cnr1* mRNA. Among them, we selected only those highly conserved among vertebrates such as miR-18, miR-190, miR-128, miR-19, miR-29, miR-181, miR-130, miR-301, miR-148, and miR-152. Afterwards, by quantitative PCR analysis, we found that miR-18, miR-128, miR-29, miR-130, miR-152, and miR-148 expression was significantly up-regulated by simvastatin as compared to control cells after 3 h of exposure (Fig. 4A). Whist, after 24 h, we observed an up-regulation of only miR-18, miR-29, miR-130, and miR-152, with the latter showing the highest up-regulation (Fig. 4B).

### AntagomiR-152 prevents simvastatin-induced cytotoxicity by rescuing CB1 expression in C2C12 cells

Based on the results described above, we wondered whether the overproduction of miRNAs targeting CB1 in skeletal muscle cells exposed to simvastatin could be a pathological mechanism contributing to cell death. To pursue this hypothesis, 24 h before simvastatin exposure, myoblasts were transiently transfected with sequences against miR-152 (antago-miR152). In parallel, myoblasts transfected with control negative antagomiR (scramble) and treated with vehicle (DMSO) or simvastatin served as control groups. After treatment for 24 h with simvastatin, cells were analyzed by FACS analysis. As expected, simvastatin significantly increased the percentage of apoptotic cells (by about 60–70%) as compared to scramble-transfected myoblasts treated with vehicle (DMSO) (Fig. 4C, D). Notably, in antago-miR152 transfected myoblasts, the number of cells in both early and/or late cell apoptosis was significantly reduced (Fig. 4C, D) along with a rescue of *Cnr1* mRNA expression at levels comparable to control cells (Fig. 4E).

### Simvastatin suppresses CB1 receptor signaling in C2C12 myoblasts

Next, to gain a better understanding of the dysfunctional role of CB1 in this experimental condition, we assessed the effect of simvastatin on the expression and function of key signal transduction events downstream of receptor activation. In our previous study, we showed that the stimulation of CB1 receptors in C2C12 cells leads to the activation of protein kinases C (PKCs), a family of serine/threonine kinases involved in numerous functions within the body including the production of DAGs (diacylglycerols) i.e., the precursors of the endocannabinoid 2-AG [15, 23]. PKCs activity is known to be regulated by phosphorylation/dephosphorylation modifications. In particular, for PKCs, the consequence of phosphorylation is an increase in their activity, while contrarily de-phosphorylation limits their enzymatic capability [23]. Using specific antibodies in western blot analysis we found that in myoblasts, simvastatin treatment significantly reduced PKC phosphorylation and, notably, ACEA prevented this effect (Supplementary Fig. 3). Similar changes induced by simvastatin were observed for ERK/pERK, an ubiquitous protein kinase known to be activated by different PKC isoforms and also downstream of CB1 receptor stimulation in different cell types [24, 25] (Supplementary Fig. 3). These data, and in particular the fact that ACEA was effective at counteracting the effect of simvastatin on PKC/ERK activation but not its effect on cell viability, indicated that the statin reduces CB1 intracellular signaling at PKCs on top, and not just as a consequence, of its down-regulation of CB1 expression, and that this effect is not sufficient per se to reduce cell viability (otherwise ACEA would have reversed simvastatin effect on cell viability too).

**Fig. 5:**
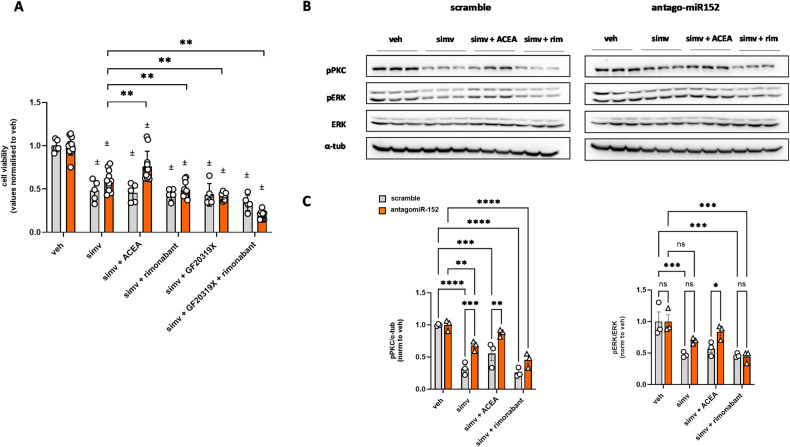
Measurement of cell viability and PKC/ERK phosphorylation in myoblasts treated with simvastatin in combination or not with CB1 drugs. **A** Bar graph with individual points reporting the cell viability measured in scramble (gray) or antagomiR-152 (orange) transfected myoblasts treated with simvastatin alone or in combination with ACEA (1 µM), rimonabant (1 µM) or GF10923X (5 µM). Each bar is the mean ± S.E.M. from at least 5 independent biological samples. * = *p* ≤ 0.05; ** = *p* ≤ 0.01 versus the indicated experimental group. **B** Representative blots showing phosphorylation of PKC (pPKC) and ERK (pERK) in C2C12 treated with simvastatin (30 µM) in combination or not with ACEA (1 µM) and rimonabant (1 µM). **C** Bar graph with individual points reporting the effect of simvastatin (30 µM) in combination or not with ACEA (1 µM) and rimonabant (1 µM) on the phosphorylation of PKC (pPKC) and ERK (pERK) in C2C12 cells, as obtained from western blots such as those represented in panel (**B**). Each bar is the mean ± S.E.M. from 3 independent biological samples. * = *p* ≤ 0.05; ** = *p* ≤ 0.01; *** = *p* ≤ 0.005 ; **** = *p* ≤ 0.0001 versus the indicated experimental groups; ± = *p* ≤ 0.005 versus the internal (scramble or simv) veh group.

At this point, to understand if the simvastatin stimulatory effect on miR-152 was necessary but not sufficient to cause a reduction of cell viability, we transfected C2C12 myoblasts with antagomir-152. In these cells, we found that simvastatin-induced cell toxicity was less marked than in negative control cells (scramble transfected) but still present, and, most importantly, that ACEA now significantly prevented this effect, rimonabant more strongly exacerbated it. This suggests that partial repression of CB1 gene expression is not the only mechanism underlying simvastatin cytotoxicity, and that, when this mechanism is impaired, other effects, such for example downstream CB1 signaling, which can be either significantly antagonized or worsened by ACEA and rimonabant, respectively, become more noticeable. This suggestion was further supported by the finding that GF109203X (5 µM), a selective blocker of PKCs, exacerbated both the effect of rimonabant plus simvastatin and that of simvastatin per se (Fig. 5A). None of these effects of GF109203X was observed in control, scramble-transfected cells (Fig. 5A). Moreover, GF109203X 5 µM alone altered neither myoblast viability phosphorylation of PKC and ERK (Supplementary Fig. 4A–C).

Finally, we measured changes in ERK and PKC phosphorylation induced by ACEA and rimonabant alone or in combination with simvastatin in control and antago-miR152 transfected cells. Similarly to the results obtained in non-trasfected cells (Supplementary Fig. 2), we found that in control scramble cells the phosphorylation of both PKC and ERK was robustly reduced following the treatment with simvastatin in a manner prevented by ACEA (as in cells treated with simvastatin and no scramble RNA), and worsened by rimonabant (Fig. 5B,C). Remarkably, in antago-miR152 transfected cells, we found that: (i) the inhibition of PKC and ERK phosphorylation by simvastatin was less pronounced compared to scramble cells, but still statistically significant; (ii) ACEA still significantly increased PKC and ERK phosphorylation in the presence of simvastatin; (iii) the effect of simvastatin was still worsened by rimonabant (Fig. 5B, C). These data suggest that, unsurprisingly, part of the effect of simvastatin on ERK and PKC phosphorylation is due to its suppression of CB1 expression, and that when the latter is lacking, CB1 agonism or antagonism can still modulate in opposite manners ERK and PKC phosphorylation. The effect of ACEA and rimonabant alone in scramble and antago-miR152 transfected cells is shown in Supplementary Fig. 5. These control data evidence how, in the absence of simvastatin, rimonabant produces no effect, whereas ACEA only causes a small enhancement of phosphorylation in scramble RNA-treated cells, which, if anything, would have rendered the CB1 agonist more, rather than less, efficacious at reversing simvastatin inhibition of ERK phosphorylation in these cells, and, therefore, is unlikely to be relevant to the results presented above. Both the lack of effect by rimonabant and the small effect of ACEA might be due to the lack of simvastatin enhancement of endocannabinoid levels under these experimental conditions.

In summary, these results suggest that simvastatin, at the concentration used here, reduces C2C12 viability via two concurrent, and partly overlapping, inhibitory effects on CB1 signaling, i.e., (1) the upregulation of miR-152 and subsequent partial suppression of CB1 expression (and, hence, signaling), and (2) the partial and direct inactivation of a signaling pathway downstream of whatever is left of CB1 receptors after such suppression. Neither of these effects appear to be per se sufficient for simvastatin to cause full myoblast death.

### Simvastatin causes alterations of CB1 signaling in the skeletal muscle of C57BL/6 J mice

**Fig. 6:**
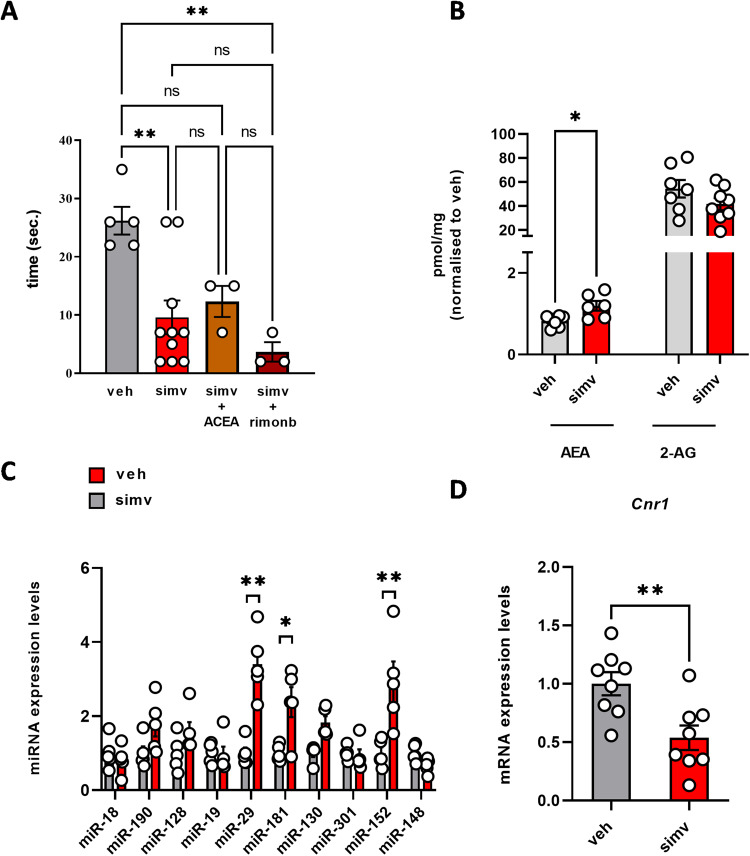
Effect of simvastatin in skeletal muscles of C57BL/6 mice. Bar graph with individual points showing the **(A)** grip test reported as the latency of mice to drop the weight **(B)** levels of AEA and 2-AG, **(C)** expression levels of miRNA targeting *Cnr1* in gastrocnemius of control and simvastatin-treated mice and **(D)** mRNA expression levels of *Cnr1* in gastrocnemius of control and simvastatin-treated mice. Each bar is the mean ± S.E.M. from five independent biological samples. * = *p* ≤ 0.05; **= *p* ≤ 0.01 versus the indicated experimental groups.

To explore whether simvastatin could cause changes in ECS activity in vivo, we used 10-week-old male C57BL/6 J mice randomized into four groups to receive a daily dose of (a) vehicle, (b) simvastatin 20 mg kg^−1^, (c) simvastatin + ACEA 2.5 mg kg^−1^ and (d) simvastatin + rimonabant 0.5 mg kg^−1^ by oral gavage for 30 days following published protocols [16, 26]. At the end of the treatment, mice were subjected to functional tests and biochemical analyses performed on dissected skeletal muscles (gastrocnemius). As expected, the grip test revealed that muscle strength in mice receiving simvastatin was significantly lower compared to their controls, whereas ACEA or rimonabant had only a slight effect (Fig. 6A). Notably, similar to C2C12 cells, in mice receiving simvastatin we found that muscle levels of the endocannabinoids AEA, but not 2-AG, were increased along with a significant up-regulation of miR-29, miR-181, and miR‐152, and reduced levels of *Cnr1* mRNA (Fig. 6B–D). In the same tissues, simvastatin drastically reduced the phosphorylation of PKC and ERK1/2, and also, as expected, increased the expression of two out of three markers of muscle toxicity such as the cardiac *TnnT2* (Troponin T2), *Myl3* (Myosin Light Chain 3) and *Fabp3* (Fatty Acid Binding Protein 3). Most crucially, ACEA significantly prevented these negative effects (Supplementary Fig. 6A–C). In summary, these results confirmed, once again, that the simple pharmacological stimulation of CB1 is not sufficient to prevent simvastatin toxicity.

### Simvastatin causes miRNA-mediated repression of CNR1 expression and functional impairment of CB1 receptor in primary human myoblasts

**Fig. 7:**
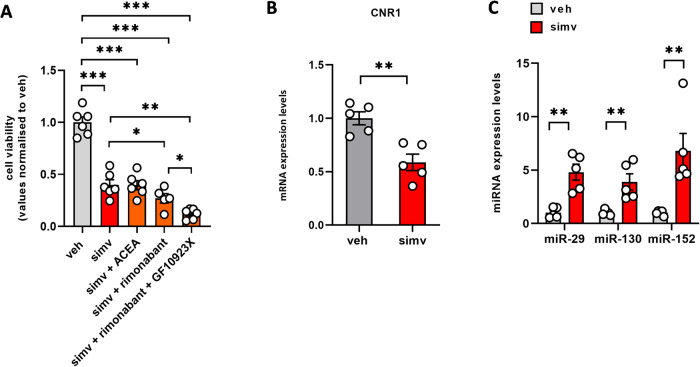
Effect of simvastatin on ECS activity in primary human myoblasts. **A** Bar graph with individual points showing the effect of simvastatin 30 µM in co-administration or not with CB1-targeting drugs (ACEA 1 µM or rimonabant 1 µM) in combination or not with GF10923X (5 µM) on cell viability measured by MTT in primary human myoblasts. **B** expression levels of CNR1 mRNA **(C)** and miRNAs targeting CNR1. Each bar is the mean ± S.E.M. from five independent biological samples. **= *p* ≤ 0.01 versus the indicated experimental groups.

Finally, the expression of miRNAs targeting CB1 and its pharmacological activity were explored in primary human myoblasts exposed to simvastatin. Also in this case, we observed that simvastatin 30 µM induced cell toxicity in more than 60% of human myoblasts in a manner not prevented by ACEA, but markedly aggravated by the co-administration of rimonabant with GF109203X. Importantly, also in primary human cells simvastatin caused a significant reduction of CNR1 mRNA expression associated with the up-regulation of miR-29, miR-130 and mostly miR-152 (Fig. 7A–C).

## Discussion

In this study, we demonstrated that simvastatin-induced myotoxicity in murine myoblasts and skeletal muscle tissues is associated with the dysregulated expression of miRNAs causing repression of the *Cnr1/*CNR1 gene expression along with parallel inhibition of downstream PKC/ERK-mediated intracellular signaling pathways coupled CB1 receptor activation.

According to recent estimates, the use of lipid-modifying agents (LMAs) to prevent cardiovascular diseases (CVDs) has grown massively worldwide, particularly over the last two decades [27]. Statins are the most prescribed class of LMAs showing the largest average growth from 2008 to 2018 [28]. The biggest turning point in the growth of statin use was in 2013 when the American College of Cardiology (ACC) and the American Heart Association (AHA) revised the existing guidelines for preventing the risk of CVDs and their complications expanding the population of patients eligible for statin therapy [29]. Therefore, according to the new ACC/AHA recommendations, statin prescription is recommended not only to those patients who have a high risk of cardiovascular events but also to those without a history of CVDs [30]. Moreover, the use of statins was further boosted due to their cholesterol-independent (pleiotropic) effects, which may include improvement of endothelial dysfunction, antioxidant properties, inhibition of inflammatory responses, and stabilization of atherosclerotic plaques [31]. Therefore, given that the patient population thought to benefit from the use of statins will undoubtedly continue to increase in the next years, unwanted adverse effects must be kept under control. Overall, the rates of severe myotoxicity with all statins are low, especially with low-to-moderate doses. However, a review article by Backes et al. reports clinical data indicating that simvastatin (80 mg daily) causes a higher incidence of myotoxicity compared with other statins [32].

The clinical spectrum of myopathies caused by statin may include myalgia, myositis, muscle pain, cramps, and less frequent rhabdomyolysis [33]. However, the exact pathophysiological mechanism(s) responsible for statin-induced myopathy is not fully known. According to a review by Gluba-Brzozka and colleagues [34], statin intolerance is mainly attributable to the perturbation of several molecular mechanisms including the reduced production of coenzyme Q10, impairment of the ubiquitin pathway, reduced production of sarcolemmal or sarcoplasmic reticular cholesterol, diminished production of prenylated proteins, disruption of calcium metabolism, induction of skeletal muscle apoptosis and mitochondrial dysfunction [34]. Alternatives to statin therapy include the use of cholesterol absorption inhibitors and bile-acid sequestrants. However, the efficacy of these drugs in reducing the levels of LDL cholesterol is much lower than that of statins. In 2015, a new class of LDL inhibitors made by human monoclonal antibodies directed against the PCSK9 enzyme, a master regulator of the LDL receptor, was approved by the US Food and Drug Administration [35]. However, the relatively high cost and the existence of conflicting data about their long-term safety profile have significantly limited the widespread use of this therapeutic tool [36]. Therefore, statins remain irreplaceable for the treatment of hypercholesterolemia and prevention of CVDs [37]. In this regard, simvastatin appears to be the best choice in terms of cost and safety, while atorvastatin and rosuvastatin are more effective to reduce cholesterol levels [38]. However, compared to atorvastatin, simvastatin is associated with a higher risk of myalgia [39].

Unbalanced ECS activity is associated with a plethora of pathological conditions affecting both the brain and peripheral organs and tissues [11, 12, 40]. For example, chronic over-activation of CB1 receptors by higher tissue levels of endocannabinoids is recognized as a pathological mechanism causing exacerbation of inflammatory responses and oxidative stress as well as obesity and its consequences, such as type 2 diabetes, liver and kidney dysfunctions and atherogenic inflammation. In some other pathological conditions, instead, transient and site-specific activation of the ECS and its activity at either CB1 or CB2 receptors takes part in the body’s compensatory responses to several insults, thus reducing symptoms and/or slowing the progression of diseases [11]. The rationale for the present study relied on published studies documenting that CB1 receptors in skeletal muscle cells regulate important metabolic pathways including insulin sensitivity and glucose uptake [17, 41]. Additionally, we demonstrated that the pharmacological stimulation of CB1 receptors by endogenous (mainly 2-AG) or synthetic (ACEA) agonists promotes the proliferation of both murine and human myoblasts and concomitantly inhibits their differentiation into mature myotubes [15]. Accordingly, using a mouse model of Duchenne muscular dystrophy (DMD), which is the most frequent and detrimental form of hereditary myopathy, we found that the selective antagonism of CB1 by rimonabant prevents locomotor impairment and promotes muscle regeneration by halting the inflammatory response [16]. These observations suggest that muscle dystrophies such as DMD may be listed among those pathological conditions that benefit from CB1 receptor antagonism rather than agonism.

Interestingly, in this study, we found, instead, that antagonism of CB1 receptors by rimonabant or AM251 worsens muscle cell toxicity induced by simvastatin, whereas direct CB1 activation by orthosteric ligands, ACEA or noladin ether, did not produce a sufficient protective effect. Of note, GAT211, a positive allosteric modulator of CB1 at least in part prevented the simvastatin-induced toxicity in C2C12 myoblasts. These findings suggest together that the impairment by simvastatin of CB1 expression is such that residual amounts of such receptors in myoblasts are present and yet not sufficient to exogenous agonizts to reverse the myotoxic effect of the drug, possibly because already fully engaged by the observed elevated levels of endocannabinoids, which, in line to their aforementioned pro-homeostatic actions, are likely to counteract this effect. Conversely, positive allosteric modulation and CB1 ligand antagonism/inverse agonism might instead better exploit the residual levels of CB1 receptors and the higher levels of its endogenous ligands to potentiate or counteract, respectively, the protective effects of such ligands at these receptors.

Most importantly, similar perturbations of endocannabinoids and CB1 signaling were found in C57BL/6 mice treated with simvastatin, thus confirming previous studies carried out with the same drugs (and supported also by histological analyses) [42, 43] and human primary myoblasts. Therefore, we believe that the reduced expression and activity of CB1 signaling caused by statins in skeletal muscle tissues might directly compromise myoblast proliferation and, hence, fusion, and, consequently, myoblast ability to terminate differentiation into mature myotubes to complete the muscle regeneration process, which is continuously ongoing even under healthy conditions.

As to the cause of simvastatin inhibitory effects on CB1 expression, we identified a specific set of miRNAs, among which miR-152 was the most up-regulated, as being potentially responsible for the suppression of CB1 gene expression in both murine and human myoblasts and mouse skeletal muscle exposed to simvastatin, and hence of the myotoxic effects of this drug. Accordingly, the antagonism of miR-152 reversed both the cytotoxic and the CB1 expression inhibitory effect of simvastatin. This is, to the best of our knowledge, the first time that CB1-targeting miRNAs are shown to underlie a pathological situation in which CB1 receptors are involved. Again in the skeletal muscle, we have also shown that, instead, suppression of CB1-targeting miRNAs, rather than their up-regulation, is responsible for some of the pathological features of DMD in vitro and vivo [44]. Additionally, in this study, we found that simvastatin not only promotes the expression of miRNA targeting CB1 gene but also inhibits the phosphorylation (and hence activation) of PKC and ERK1/2, which are major effectors of the CB1 receptor signaling pathway in C2C12 myoblasts. Since PKC/ERK phosphorylation inhibition by simvastatin, unlike its impairment of cell viability, was efficaciously counteracted by CB1 agonism with ACEA, we surmise that this effect of the statin is not just a mere consequence of simvastatin-induced *Cnr1* mRNA and protein down-regulation, and confirms that the latter effect alone does not lead to complete suppression of CB1 signaling. Our pharmacological data with a CB1 receptor agonist, antagonist and positive allosteric modulator, as well as with a selective pan-PKC inhibitor, under conditions in which the stimulatory effect of simvastatin on miR-152 is either allowed or denied by cell transfection with an antago-miRNA, clearly suggest that: (1) simvastatin does neither completely erase *Cnr1* expression, as already mentioned above, nor CB1-PKC/ERK signaling, (2) simvastatin-induced elevation of endocannabinoid levels in myoblasts partly counteracted the cytotoxic effect, (3) the restoration of both CB1 expression and signaling is key to prevent statin-induced cell toxicity, as demonstrated by the fact that ACEA exerts a statistically significant protective action on this effect only in antago-miR152 transfected cells. Indeed, both effects are necessary, but neither of them is sufficient alone for the full inhibition of cell viability by the statin, probably because: (1) both effects are not fully exerted by simvastatin at the concentration used, and (2) simvastatin also triggers in myoblasts the elevation of endogenous CB1 ligands, which exploit residual CB1 receptors and their coupling to a non-fully inhibited PKC/ERK pathway to tonically counteract the effect of the statin from within the cells. This hypothesis explains also why pharmacological tools that act, in opposing manners, through endogenous ligands tonically activating CB1, i.e., GAT211 and rimonabant (Fig. 2B and Supplementary Fig. 2), or its downstream signaling pathway, i.e., GF109203X (Fig. 5A), affect simvastatin actions, whereas ACEA, which acts on CB1 regardless of the increased tone of endogenous ligands, does not counteract simvastatin action unless the antago-miR152 is overexpressed in cells (Fig. 5B, C), i.e., when the effect of the statin depends uniquely on the event that is counteracted by the CB1 agonist.

In conclusion, we propose that CB1 receptors may represent a novel target for adjuvant therapies to prevent statin-induced myopathies and their toxic effects on muscle precursor cells. Although we could not reverse nor ameliorate here simvastatin-induced myotoxicity with CB1 agonizts (which would have little therapeutic use anyway, due to their unwanted psychotropic effects), our results, based on the worsening effects of rimonabant in particular observed when given in co-administration with the PKC inhibitor GF109203X, open the way to CB1 receptor signaling as a promising target against statin-induced myotoxicity. Conversely, the proposed use of CB1 receptor antagonists as anti-atherogenic drugs [45] in combination with statins might need to be rediscussed based on our present results.

Last but not least, we studied here only the effect of the acid form of simvastatin. A research article by Skottheim et colleagues demonstrates that in skeletal muscle cells, simvastatin lactone (a pro-drug) shows about 37-fold higher potency than simvastatin acid in inducing cell toxicity [46]. Thus, future investigations are needed also to distinguish the effect of the acid *vs* lactone form of simvastatin on CB1 expression and signaling.

## Materials and methods

### Animal model and drug treatment

The Animal Study Protocol (IACUC; 481/2023) was approved by the Italian Ministry of Health and Ethics Committee for the use of experimental animals being conformed to guidelines for the safe use and care of experimental animals following the Italian D.L. no. 116 of 27 January 1992 and associated guidelines in the European Communities Council (86/609/ECC and 2010/63/UE). In this study, 5 weeks old male C57BL/6 mice were purchased from Charles River Laboratories (Milan IT). All mice were housed in ventilated cages with a 12-h light-dark cycle and received standard mouse chow (Harlan Teklad) and water *ab libitum*. Animals were randomly subdivided into four groups receiving (a) vehicle (dimethyl sulfoxide – DMSO 0.03%, Cat# 276855 Merk) dissolved in water; (b) simvastatin 20 mg kg^−1^ (Cat# S6196 Merk); (c) ACEA 2.5 mg kg^−1^ and (d) rimonabant 0.5 mg kg^−1^ following published procedures [16, 26].

Each drug (or vehicle) was administered by oral gavage for 30 days [26]. The experimenter(s) performing the treatments and locomotor testing was blind to the genotype and treatment. Animals were anaesthetized with enflurane inhalation before being sacrificed. All efforts were made to minimize the number of animals used and their suffering.

### Grip strength test

To test the forelimb strength, control and simvastatin-treated mice were handled by the base of the tail and allowed to grasp four weights of 20, 33, 46, and 59 g. If the mouse dropped the first weight (20 g) in less than 3 s (s), we tried the same weight again a maximum of three times. If the mouse held it for 3 s, then we tried it on the next heaviest weights. The mouse was assigned the maximum time/weight achieved. The final total score is calculated as the product of the number of links in the heaviest chain held for the full 3 s, multiplied by the time (s) it is held [47].

### Cell culture and reagents

Murine C2C12 myoblasts were propagated in a growth medium (GM) composed of Dulbecco’s modified Eagle’s medium (cat# 11995065; Life Technologies) supplemented with 10% fetal bovine serum (FBS, cat# 16000044; Life Technologies), 5000 U/ml penicillin plus 5000 µg/ml streptomycin (cat# 15070063; Life Technologies), and 1% L-glutamine (cat# A2916801; Life Technologies). Proliferating C2C12 cells were differentiated into myotubes following their exposure in a differentiation medium (DM) composed of Dulbecco’s modified Eagle’s medium supplemented with 2% horse serum heat-inactivated (cat# 26050070, Merk) for four days [47]. C2C12 cells were routinely tested for mycoplasma contamination using PCR Mycoplasma Test Kit (cat# MP0050).

Primary human myoblasts were provided by Innoprot (Cat# P10977; Bizkaia-Spain) and propagated in a growth medium (GM) recommended by the same company (Skeletal Muscle Cell Medium, cat. no. P60124). Arachidonyl-2′-chloroethyl amide hydrate (ACEA) was from Merk (cat# A9719); Noladin ether (cat# 1411), Rimonabant/SR141716A (cat# 0923), AM251 (cat# 1117/1) and GF10923 (cat# 0741) were purchased from Tocris (UK). GAT211 (cat# SML1926) and simvastatin (cat# S6196) were purchased from Merk (IT). Before use, simvastatin was activated following the protocol provided by the company.

### Cell transfection and antagomir overexpression

Myoblasts at a confluency of 60% were transiently transfected with antagomiR-152 (cat# MIN0000162, Quiagen Italy) using Lipofectamine 2000 (cat# 11668019, Thermo Fisher Italy) according to the manufacturer’s instructions. After 24 h, C2C12 myoblasts were treated with simvastatin for 24 h and then subjected to FACS analysis.

### RNA extraction and quantitative PCR (qPCR)

Total RNA was isolated from C2C12 and human myoblasts, or muscle tissues by use of TRIzol Reagent (cat# 15596018, Life Technology), reacted with DNase-I (cat# AMPD1 Merk) for 15 min at room temperature, followed by spectrophotometric quantification. Subsequently, the RNA integrity number (RIN) for each RNA sample was analyzed on the Agilent 2100 bioanalyzer. Purified RNA was reverse-transcribed by the use of the iScript cDNA Synthesis Kit (cat# 1708841 Biorad). Total miRNAs isolation was performed using RNeasy Mini Kit (cat# 217004, Qiagen). Reverse transcription of total miRNAs was performed using miScript II RT Kit (cat# 218161, Qiagen).

Quantitative PCR (qPCR) was carried out in a real-time PCR system CFX384 (Bio-Rad) using the SYBR Green PCR Kit (cat# 1725274, Bio-Rad for mRNAs; cat# 218073, Quiagen for miRNAs) detection technique and specific primer sequences [15]. Primer sequences for miRNA and/or antagomiRNA152 were provided by Qiagen. Quantitative PCR was performed on independent biological samples ≥5 for each experimental group. Each sample was amplified simultaneously in quadruplicate in a one-assay run with a nontemplate control blank for each primer pair to control for contamination or primer-dimer formation, and the cycle threshold (Ct) value for each experimental group was determined. The housekeeping genes ribosomal protein S16 and U6 (RNU6‑1) were used to normalize the Ct values, using the 2^^−ΔCt^ formula. Differences in mRNAs and miRNAs content between groups were expressed as 2^^−ΔΔCt^, as previously described [47]. The primer sequences used were: *murine CB1 forw* 5’-GGGCACCTTCACGGTTCTG-3’; *murine CB1 rev 5’-*GTGGAAGTCAACAAAGCTGTAGA-3’; *murine S16 forw* 5’-CTGGAGCCTGTTTTGCTTCTG-3’; *murine S16 rev* 5’-CTGGAGCCTGTTTTGCTTCTG-3’; *human CB1 forw* 5’-TCGGACGCAAGAAGACAGCGA-3’; *murine CB1 rev 5’-*GTGGAAGTCAACAAAGCTGTAGA-3’; *human S16 forw* 5’-TCGGACGCAAGAAGACAGCGA-3’; *human* S16 rev 5’-AGCGTGCGCGGCTCAATCAT-3’; *murine Fabp3 forw* 5’-ACCAAGCCTACTACCATCATCG-3’; *murine Fabp3 rev 5’-*CCTCGTCGAACTCTATTCCCAG-3’; *murine TnnT-2 forw* 5’-CAGAGGAGGCCAACGTAGAAG-3’; *murine TnnT-2 rev 5’-*CTCCATCGGGGATCTTGGGT-3’; *murine Myl3 forw* 5’-TGCCTCCAAGATTAAGATCGAGT-3’; *murine TnnT-2 rev 5’-*CTCTGCCTGGGTAGGATTCTG-3’. Primer sequences for miRNAs were provided by Qiagen (IT).

### miRNA target prediction

Bioinformatic analysis to predict putative miRNA target sites within the 3’UTR region of both human and murine CB1 genes was performed using the free software TargetScan (http://www.targetscan.org/vert_80/).

### Western blot

Total protein from control and statin-treated C2C12 cells or muscle tissue were exacted using a 1x TNE buffer [50 mm Tris–HCl (pH 7.4); 100 mM NaCl. 0.1; mM EDTA) plus 1% (v/v) Triton X-100 (cat# T8787, Sigma-Aldrich) and protease inhibitor (cat# P8340, Sigma-Aldrich). Lysates were kept in an orbital shaker incubator at 220 rpm at 4°C for 30 min and then centrifuged for 15 min at 13,000 g at 4 °C. The supernatants were transferred to tubes and quantified by DC Protein Assay (cat# 5000116, Bio-Rad, Milan, Italy). Subsequently, protein samples (60–80 μg of total protein) were heated at 70 °C for 10 min in 1X LDS Sample Buffer (cat# B0007, Life Technology) plus 1X sample reducing agent (cat# B0009, Life Technology) and loaded on 10% Bis-Tris Protein Gels (cat# NW00102BOX, Life Technology) and then transferred the membrane using Trans-Blot Turbo Mini 0.2 µm PVDF Transfer Packs (cat# 1704156 Bio-Rad). The primary antibodies used were: (a) rabbit anti-CB1 (cat# Y409605, ABM Canada); (b) rabbit anti phospho-PKC (cat# 190D10, Cell Signaling USA); (d) rabbit anti phospho-ERK1/2 (cat# 9101, Cell Signaling USA) and (e) rabbit anti-ERK1/2 (cat# 4695, Cell Signaling USA); (f) mouse anti α-tubulin antibody (1D4) (cat#. T6199; Merk). Reactive bands were detected by Clarity Western ECL Substrate (cat# 1705061 Bio-Rad). The intensity of bands was analyzed on a ChemiDoc station with Quantity-one software (Biorad, Segrate, Italy).

### 3-(4,5-Dimethylthiazol-2-yl)-2,5- diphenyltetrazolium bromide (MTT) assay

C2C12 cells and primary myoblasts were seeded in 24-well culture plates (25000 cells/well). Myoblasts were allowed to attach overnight or to differentiate into myotubes for 4 days before starting the treatment with the compounds of interest. Cells were then treated for 24 h with the test compounds. Afterwards, cell media was replaced by MTT solution (0.5 mg/mL, pH 7.4) for 3 h at 37 °C. Formazan precipitates formed were dissolved using an isopropanol solution and absorbance was recorded at 595 nm using a Promega GloMax Plate Reader.

### Caspase3/7 assay

C2C12 myoblasts (2000 cells/well) were seeded in 96-well black plates. After 24 h, cells were treated for 24 h with compounds of interest. Caspase3/7 activation was determined using the Caspase-Glo 3/7 Assay System (cat# G8090, Promega). Luminescence was measured with a Promega GloMax Plate Reader. Chemiluminescent signals were acquired using a ChemiDoc station (Biorad, Segrate, Italy).

### Flow cytometry and cell cycle analysis

Cell apoptosis was analyzed using an Annexin V-FITC kit purchased from BD Pharmingen (San Diego, CA, USA) according to the manufacturer’s instructions. C2C12 cells were seeded in 6 well plates and allowed to attach overnight. The cells were treated with simvastatin (30 μM) in the presence or absence of Rimonabant (1 µM) or ACEA (1 µM) for 24. After this time, cells were collected and washed twice with PBS. Samples were then taken to determine baseline and drug-induced apoptosis by Annexin V-FITC/Propidium Iodide (PI) (Beckman Colter; Brea, CA) double staining or PI staining and flow cytometry analysis using a FACSCanto II 6-color flow cytometer (Becton Biosciences, San Jose, CA), as described previously [48]. To detect early and late apoptosis, both adherent and floating cells were harvested together and resuspended in annexin V binding buffer (10 mM HEPES/NaOH pH 7.4, 140 mM NaCl, 2.5 mM CaCl2) at a concentration of 106 cells/mL. Subsequently, 5 μL of FITC-conjugated Annexin V and 5 μL of PI were added to 100 μL of the cell suspension (105 cells). The cells were incubated for 15 min at room temperature in the dark. Finally, 400 μL of annexin V binding buffer was added to each tube. A minimum of 50,000 events for each sample were collected and data were analyzed using FlowJo v10 software (Tree Star, Ashland, OR, USA).

### Measurement of endocannabinoids

Murine or human skeletal muscle cells were collected and sonicated in a solution containing 50 mmol/L chloroform/methanol/cell media (2:1:1, vol/vol). Muscle tissues were first dounce-homogenized in a solution containing 50 mmol/L chloroform/methanol/Tris·HCl, pH 7.5 (2:1:1, vol/vol) and then sonicated for 8 min. After sonication, internal standards [[2H]8 anandamide (AEA) 10 pmol; [2H]5 2-arachidonoylglycerol (2-AG)] were added to the solutions. The organic phase containing lipids was dried down, weighed, and purified by open-bed chromatography on silica gel. Fractions were obtained by eluting the column with 99:1, 90:10, and 50:50 (vol/vol) chloroform/methanol. The 90:10 fraction was used for AEA and 2-AG quantification by liquid chromatography–atmospheric pressure chemical ionization–mass spectrometry by using a Shimadzu HPLC apparatus (LC-10ADVP) coupled to a Shimadzu (LCMS2020) quadrupole mass spectrometry via a Shimadzu atmospheric pressure chemical ionization interface as previously described [49]. The amount of endocannabinoids in both cells and tissues, quantified by isotope dilution with the above-mentioned deuterated standards, is reported as pmol/mg of the total amount of lipids extract.

### Statistical analysis

Data and statistical analysis comply with the recommendations on experimental design and analysis in pharmacology [50]. Data are expressed as means ± SEM of values. All data were analyzed by one-way or two-way ANOVA using GraphPad Prism 10 (GraphPad Software, La Jolla, CA, US). Significance was determined as *p* < 0.05. GraphPad Prism log (agonist) *vs* response-variable slope (four parameters) non-linear regression analysis was used to determine the effect of simvastatin in myoblast and myotubes cells. The sample size was calculated according to ref. [51].

## Supplementary information

Supplementary Figures legend

Supplementary Figure 1

Supplementary Figure 2

Supplementary Figure 3

Supplementary Figure 4

Supplementary Figure 5

Supplementary Figure 6

uncropped western blot

co-authors agreement

## Data Availability

The experimental data sets generated and/or analyzed during the current study are available from the corresponding author upon reasonable request. No applicable resources were generated during the current study.
