# Supplementary material for: Dysfunctional endocannabinoid CB1 receptor expression and signaling contribute to skeletal muscle cell toxicity induced by simvastatin
Source: Cell Death Dis. 2023 Aug 23;14(8):544. doi: 10.1038/s41419-023-06080-9 (PMC10447569; doi:10.1038/s41419-023-06080-9)
Supplement: Supplementary file 1 — Supplementary Figures legend [file 41419_2023_6080_MOESM1_ESM.docx]

**Supplementary Figure 1: Effect of simvastatin on ECS activity in C2C12 myotubes.** Bar graph with individual points showing the levels of AEA and 2-AG **(A)** and the expression levels of *Cnr1* mRNA **(B)** in C2C12 myotubes exposed to simvastatin 30 µM for 24 h. Each bar is the mean ± S.E.M. from five independent biological samples. * = p≤0.05; ** = p≤0.01; versus the indicated experimental group.

**Supplementary Figure 2: Cell viability measured in C2C12 myoblasts exposed to simvastatin in the presence of orthosteric and allosteric CB1 agonists/antagonists.** Measurement of cell viability using MTT assay in myoblasts exposed to simvastatin (30 µM) for 24h in the presence or absence of the selective CB1 full agonists (ACEA and noladin ether, 1 µM) or antagonists (rimonabant and AM251, 1 µM) at 3 and 24h. **(B)** Concentration-dependent effect of the positive allosteric modulator (PAM) of CB1, GAT211 in myoblasts exposed to simvastatin (30 µM) for 24h. Each bar is the mean ± S.E.M. from five independent biological samples. **** = p≤0.001; *** = p≤0.001; ** = p≤0.07 between the indicated experimental groups.

**Supplementary Figure 3: Effect of simvastatin in combination or not with CB1-targeting pharmacological tools on PKC and ERK phosphorylation in C2C12 myoblasts. (A)** Representative blots showing changes in the expression and phosphorylation of PKC and ERK in C2C12 myoblasts treated with simvastatin (30 µM) in the presence or absence of ACEA (1 µM). **(B)** Bar graph with individual points showing quantification of western blot results. Each bar is the mean ± S.E.M. from 3 independent biological samples. * = p≤0.05; *** = p≤0.001 versus the indicated experimental groups.

**Supplementary Figure 4: Effect of GF109203X on viability and PKC and ERK phosphorylation in C2C12 myoblasts. (A)** Bar graph with individual points showing the viability of C2C12 myoblasts exposed to increasing concentrations of GF109203X for 24 h measured using the MTT assay. Each bar is the mean ± S.E.M. from 3 independent biological samples. *** = p≤0.006 versus the indicated experimental groups. **(B)** Representative blots showing changes in the expression and phosphorylation of PKC and ERK in C2C12 myoblasts treated with vehicle (DMSO) or GF109203X (5 µM) for 24 **(C)** Bar graph with individual points showing quantification of western blot results.

**Supplementary Figure 5: Effect of ACEA and rimonabant on PKC and ERK phosphorylation in C2C12 myoblasts (A)** Representative blots showing changes of PKC and ERK phosphorylation measured in C2C12 myoblasts transfected with negative control scramble and antago-miR152 sequences treated with ACEA (1 µM) and rimonabant (1 µM) for 24 h. **(B)** Bar graph with individual points showing quantification of western blot results. Each bar is the mean ± S.E.M. from 3 independent biological samples. * = p≤0.01; ** = p≤0.005 versus the indicated experimental groups.

**Supplementary Figure 6: Effect of simvastatin in combination or not with CB1 drugs on PKC and ERK phosphorylation and expression of myotoxicity markers.** **(A)** Representative blots showing changes in the expression and phosphorylation of PKC and ERK in gastrocnemius of control and simvastatin-treated mice in coadministration with ACEA and rimonabant; **(B)** Bar graph with individual points showing quantification of western blot results. **(C)** Bar graph with individual points showing the levels of *Myl3*, *Fabp3* and *TnnT2* in gastrocnemius of control and simvastatin-treated mice in coadministration with ACEA and rimonabant. Each bar is the mean ± S.E.M. from 3 independent biological samples. * = p≤0.05; ** = p≤0.01 versus the indicated experimental group.
