# Supplementary figures and images for: Dysfunctional endocannabinoid CB1 receptor expression and signaling contribute to skeletal muscle cell toxicity induced by simvastatin
Source: Cell Death Dis. 2023 Aug 23;14(8):544. doi: 10.1038/s41419-023-06080-9 (PMC10447569; doi:10.1038/s41419-023-06080-9)

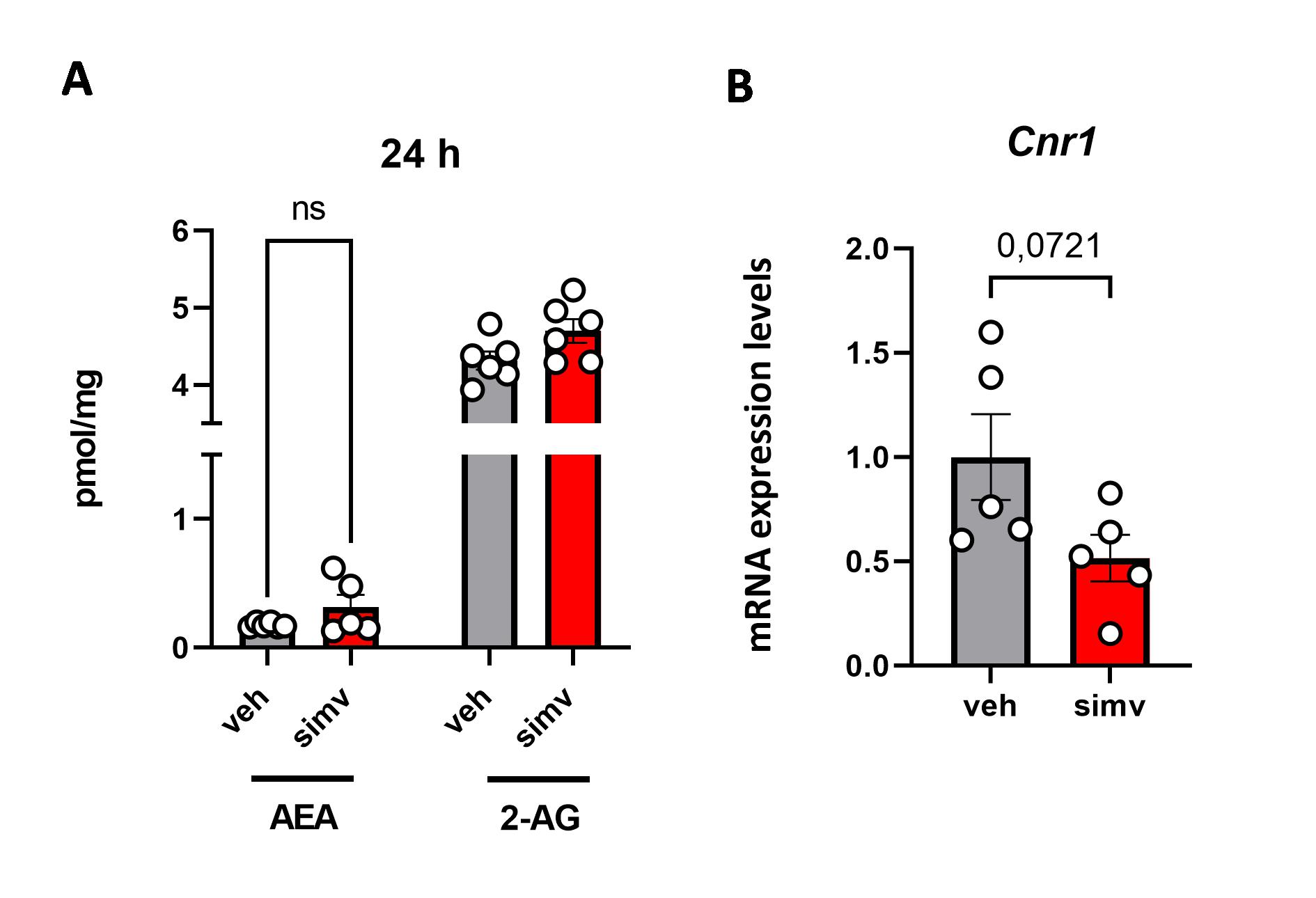

Supplement: Supplementary file 2 — Supplementary Figure 1 [file 41419_2023_6080_MOESM2_ESM.tif]

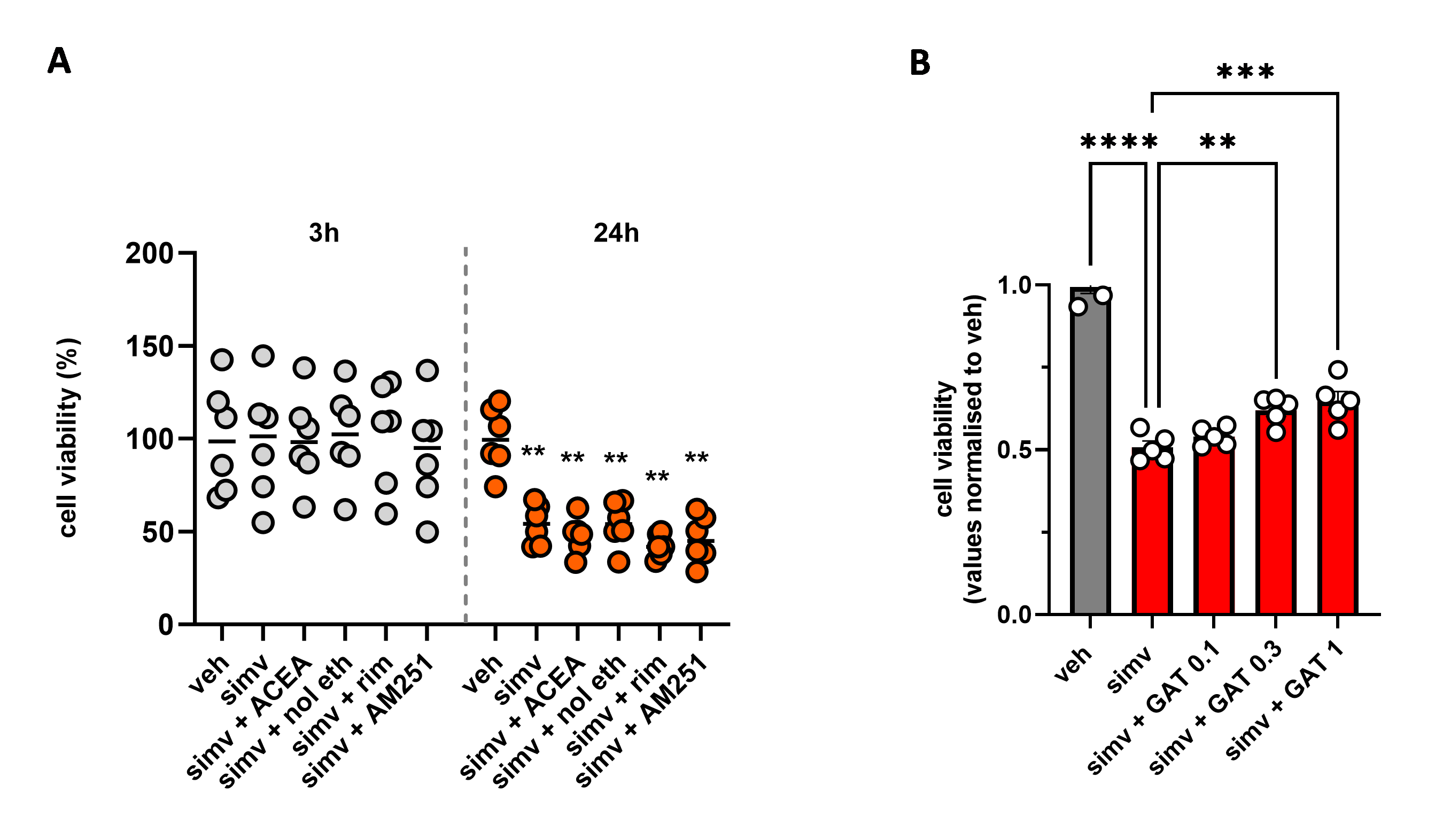

Supplement: Supplementary file 3 — Supplementary Figure 2 [file 41419_2023_6080_MOESM3_ESM.tif]

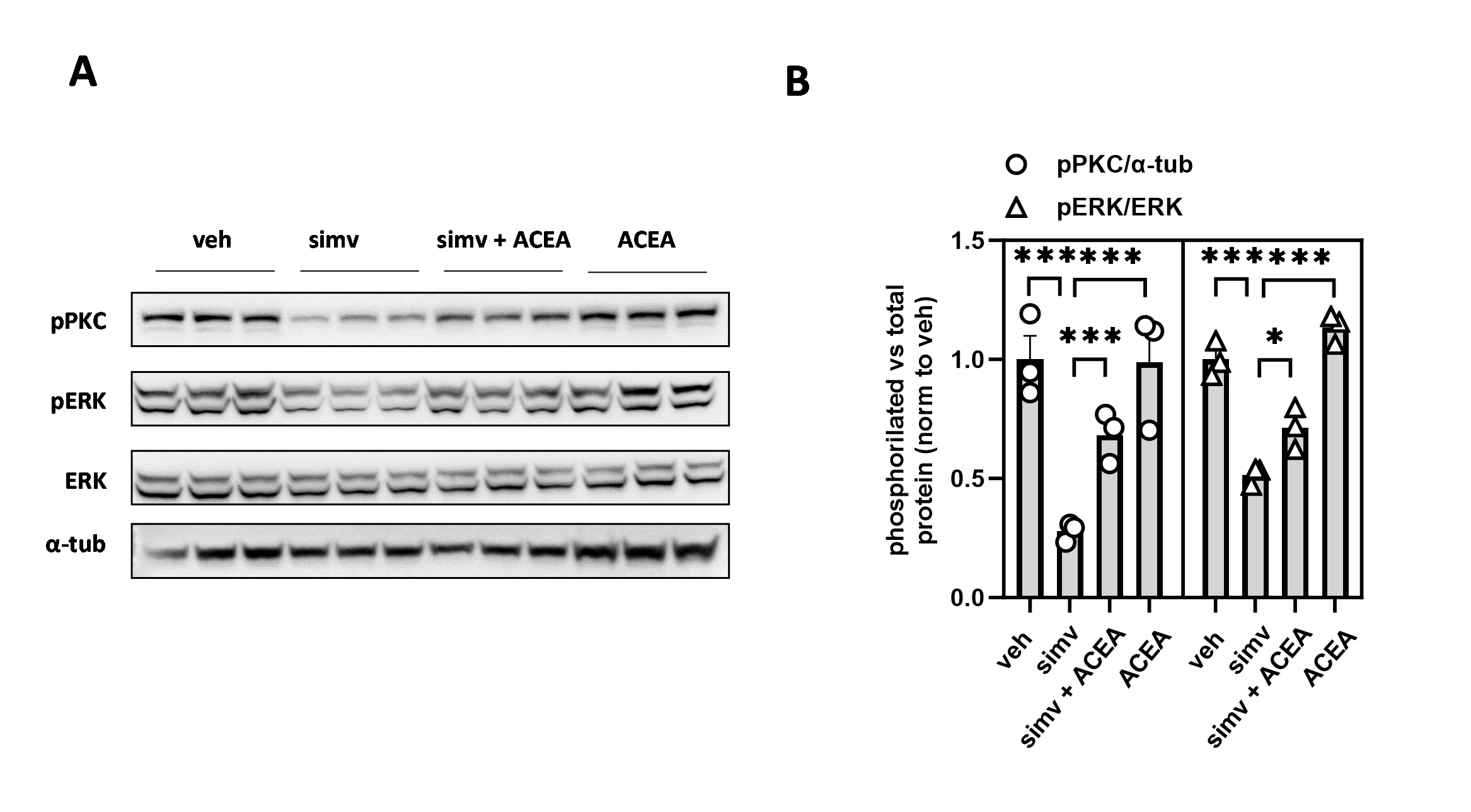

Supplement: Supplementary file 4 — Supplementary Figure 3 [file 41419_2023_6080_MOESM4_ESM.tif]

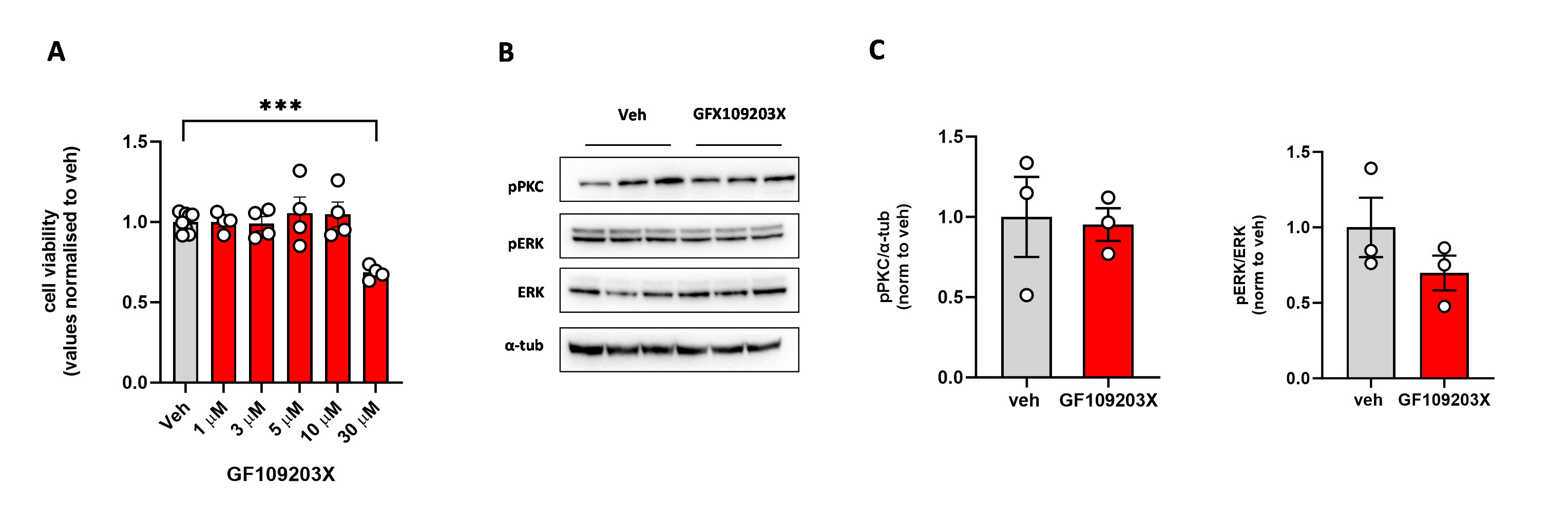

Supplement: Supplementary file 5 — Supplementary Figure 4 [file 41419_2023_6080_MOESM5_ESM.tif]

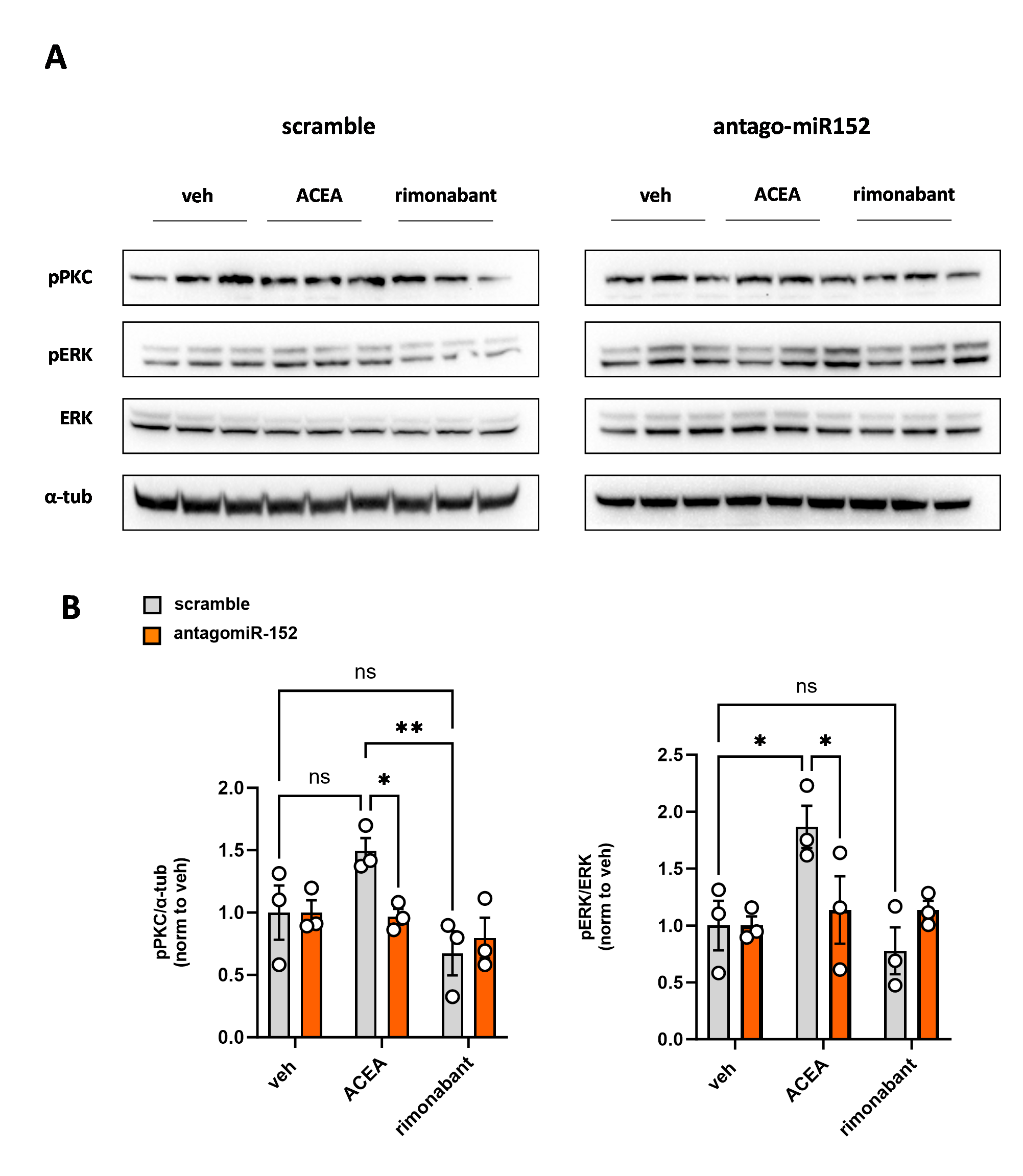

Supplement: Supplementary file 6 — Supplementary Figure 5 [file 41419_2023_6080_MOESM6_ESM.tif]

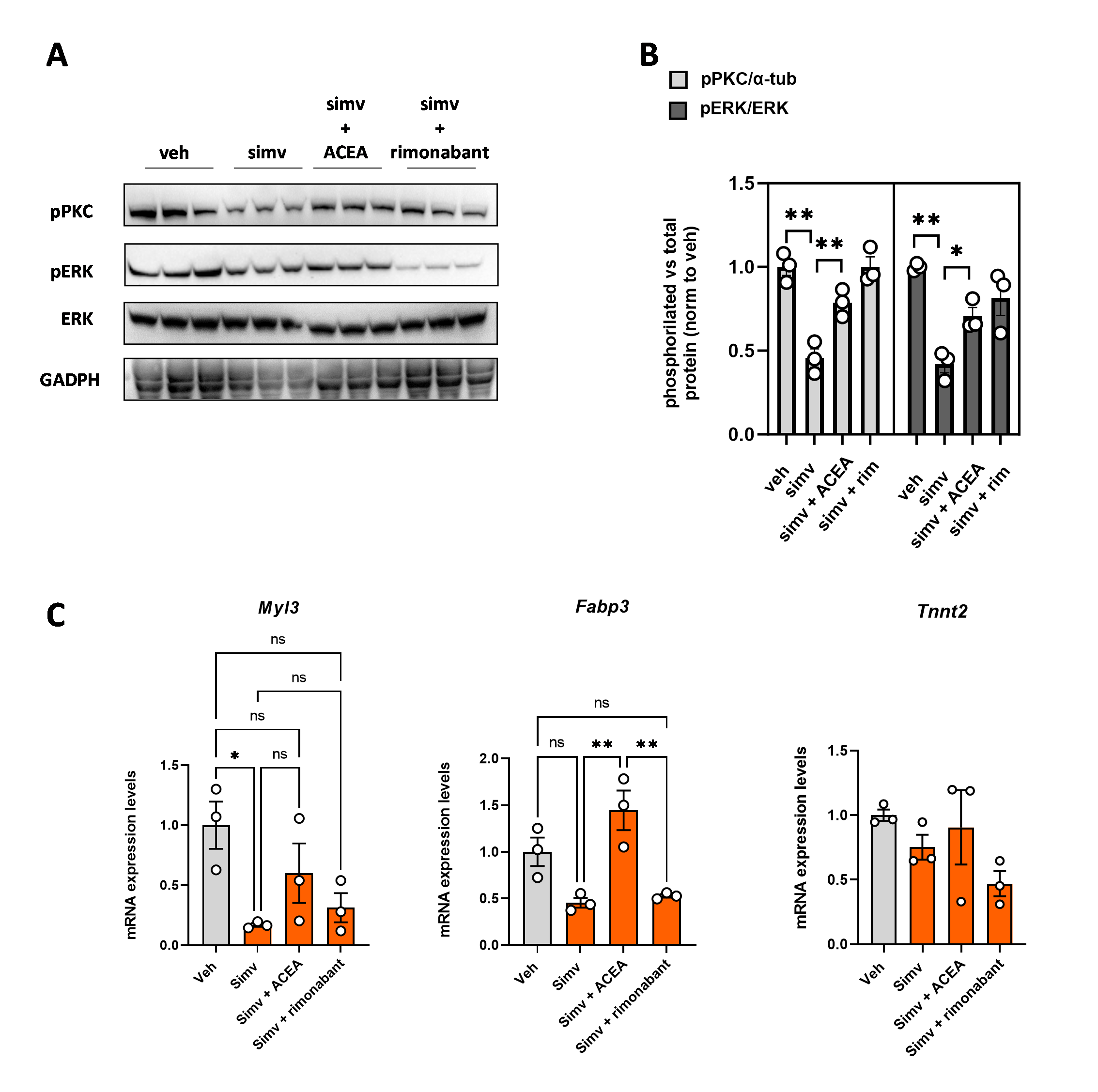

Supplement: Supplementary file 7 — Supplementary Figure 6 [file 41419_2023_6080_MOESM7_ESM.tif]
