# Supplementary material for: Dysfunctional endocannabinoid CB1 receptor expression and signaling contribute to skeletal muscle cell toxicity induced by simvastatin
Source: Cell Death Dis. 2023 Aug 23;14(8):544. doi: 10.1038/s41419-023-06080-9 (PMC10447569; doi:10.1038/s41419-023-06080-9)
Supplement: Supplementary file 8 — uncropped western blot [file 41419_2023_6080_MOESM8_ESM.pdf]

Raw blots shown in Fig. 1

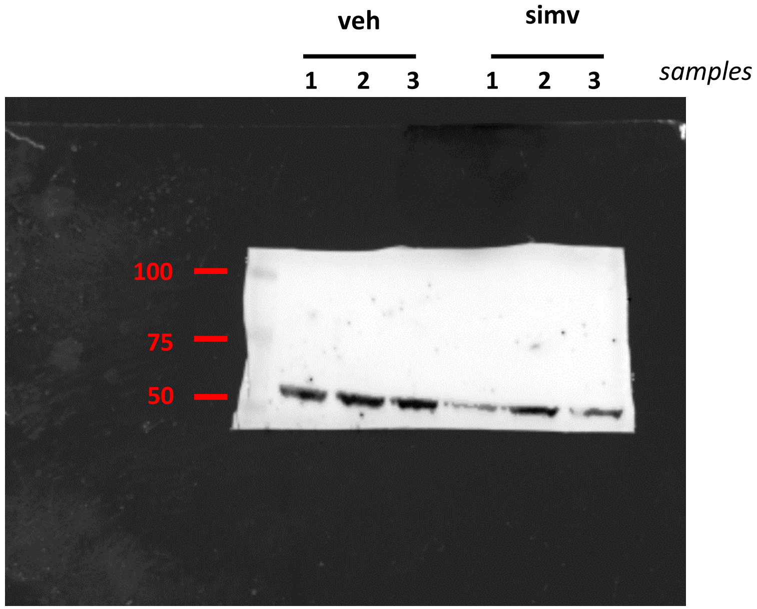

CB1 Predicted MW 53 Kda

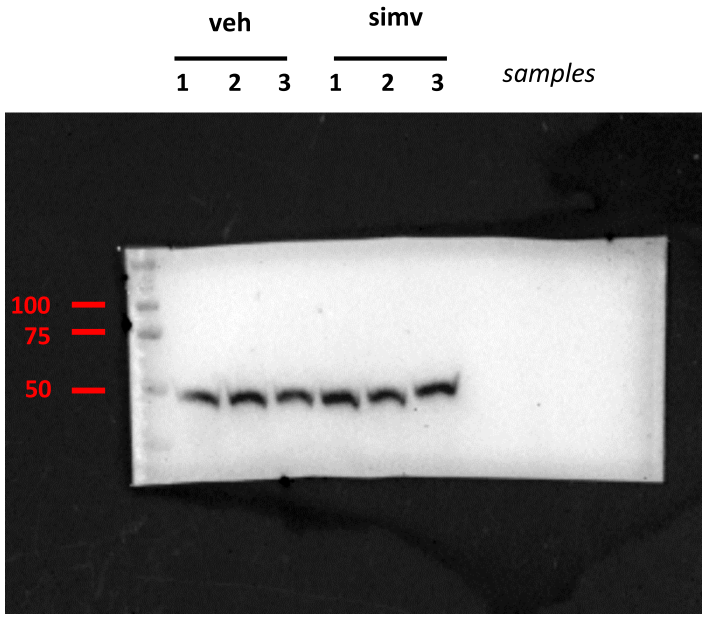

α-tubulin predicted MW 50-55 Kda

Raw blots shown in Fig. 5

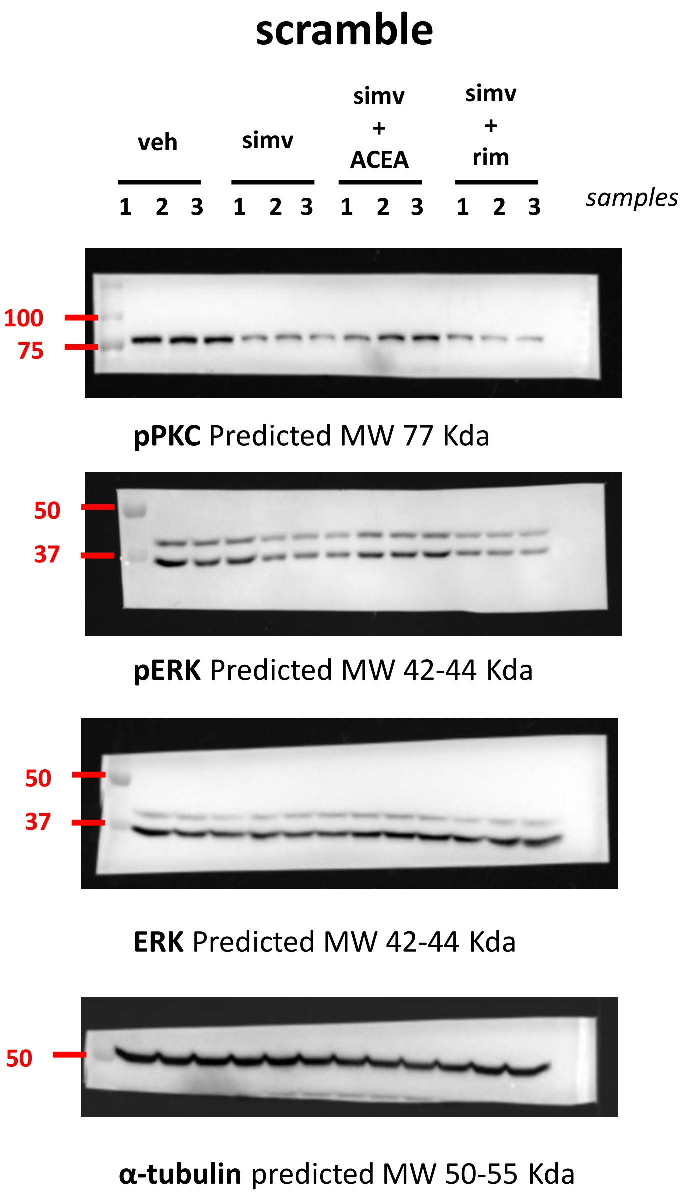

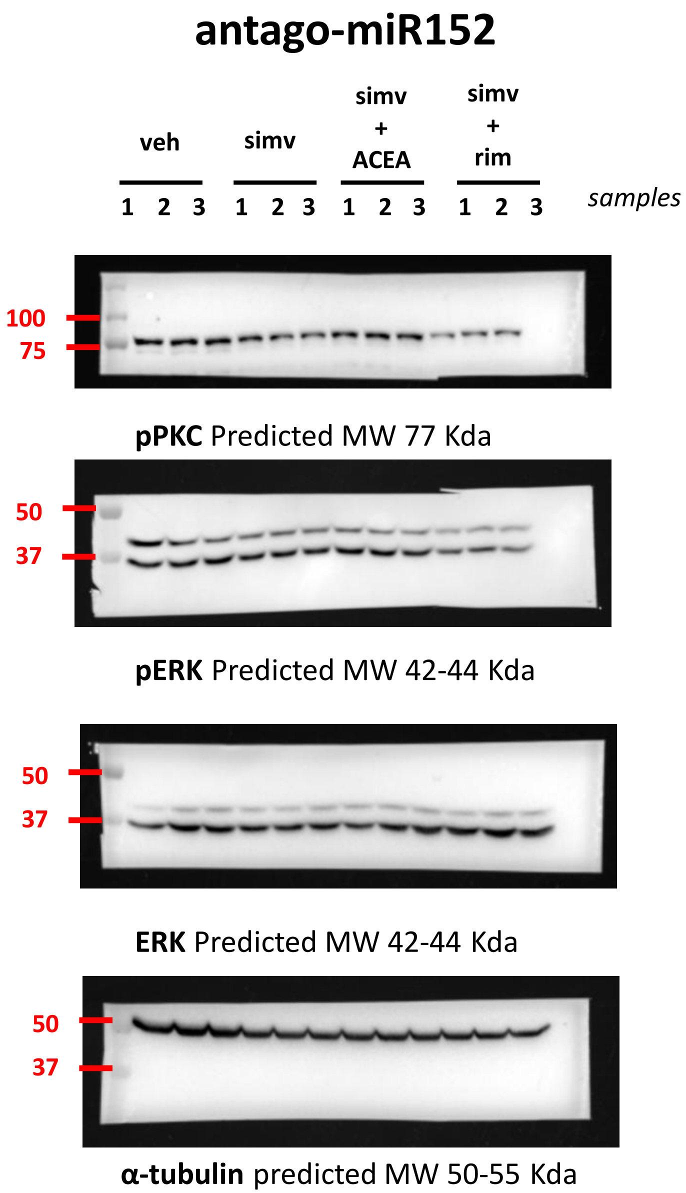

Raw blots shown in Supplementary Fig. 3

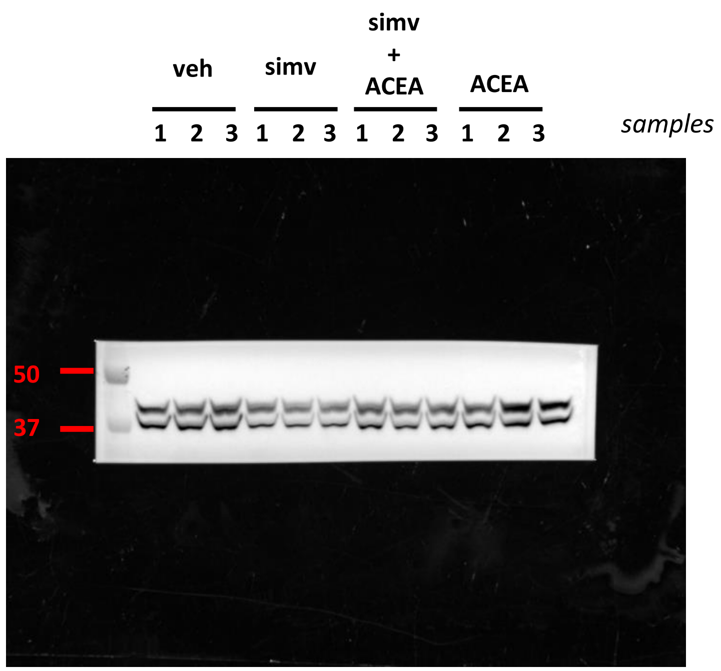

pERK Predicted MW 42-44 Kda

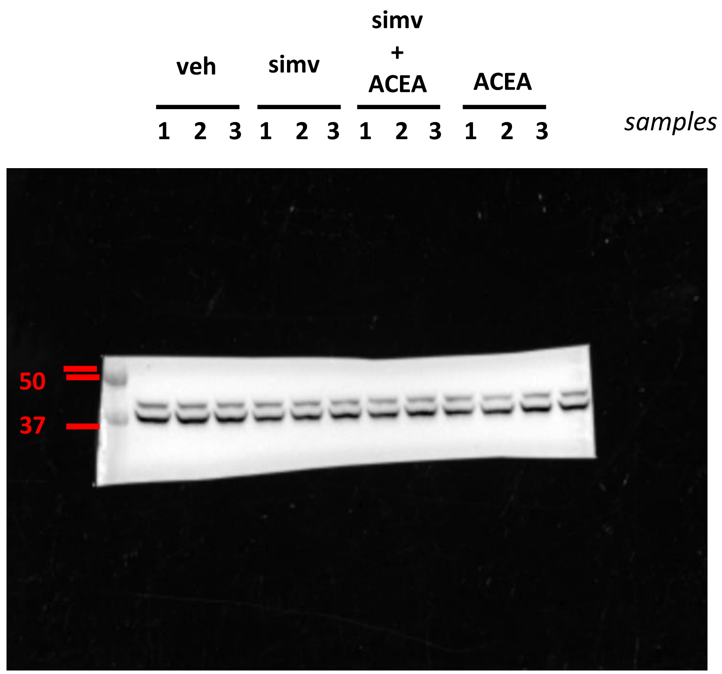

ERK Predicted MW 42-44 Kda

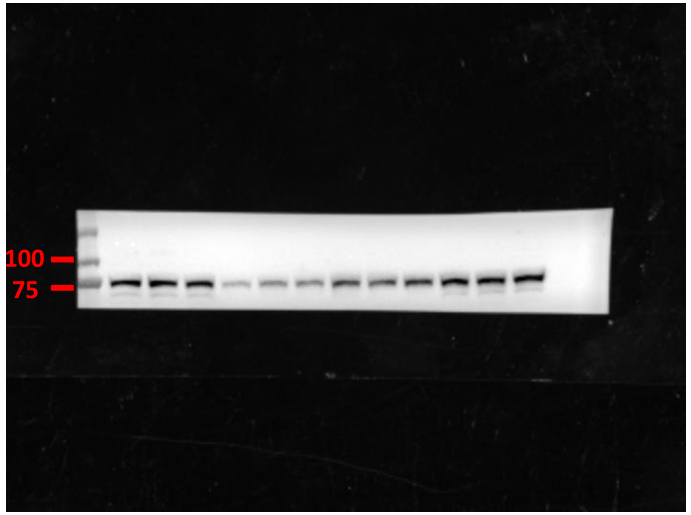

pPKC Predicted MW 77 Kda

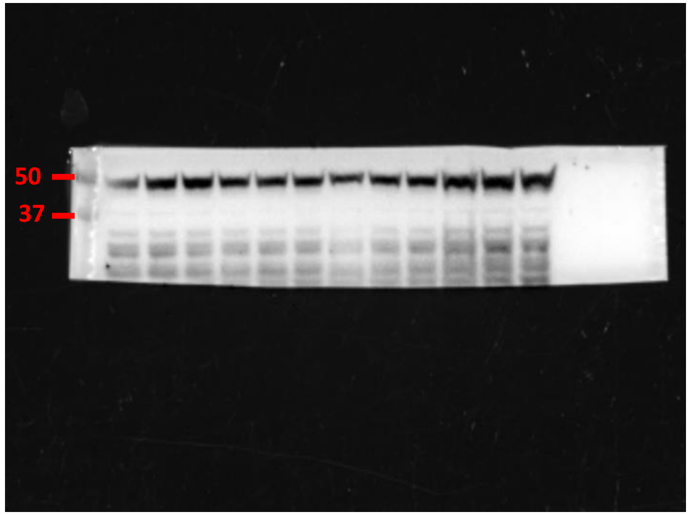

α-tubulin predicted MW 50-55 Kda

Raw blots shown in Supplementary Fig. 4

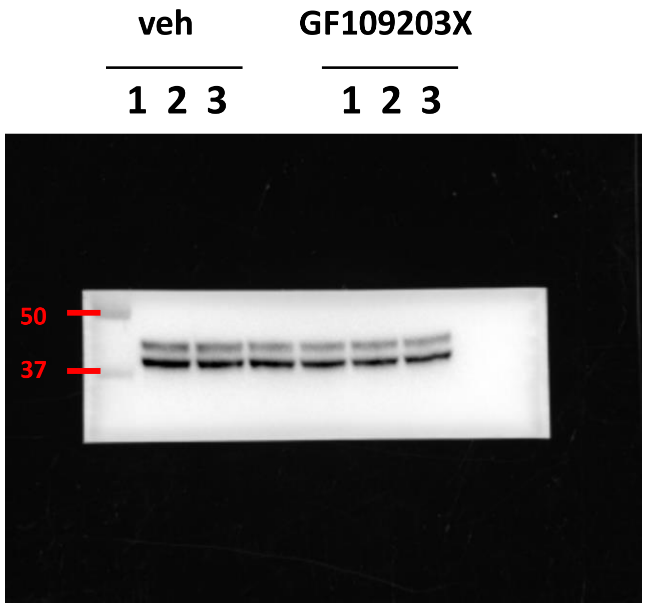

pERK Predicted MW 42-44 Kda

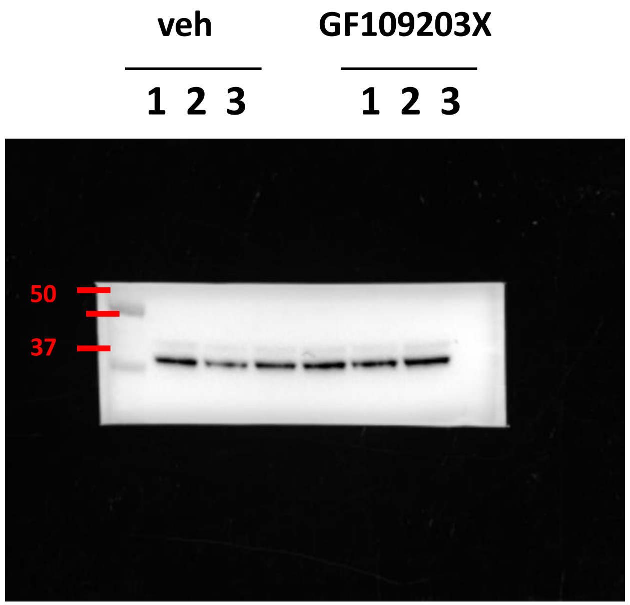

ERK Predicted MW 42-44 Kda

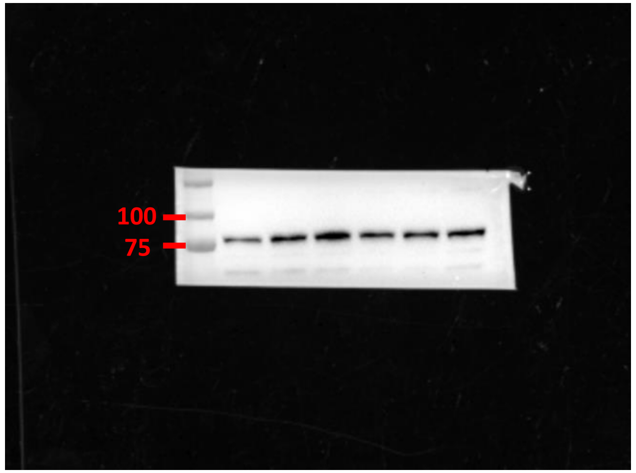

pPKC Predicted MW 77 Kda

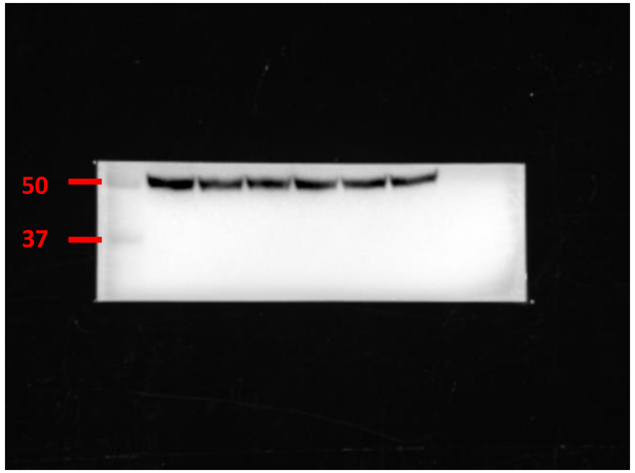

α-tubulin predicted MW 50-55 Kda

Raw blots shown in Supplementary Fig. 5

scramble

veh ACEA rim

1 2 3 1 2 3 1 2 3 *samples*

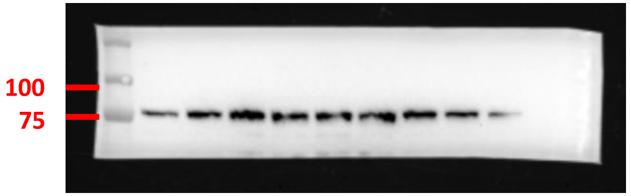

pPKC Predicted MW 77 Kda

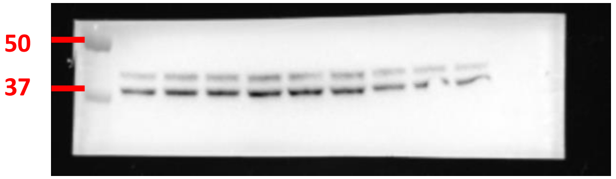

pERK Predicted MW 42-44 Kda

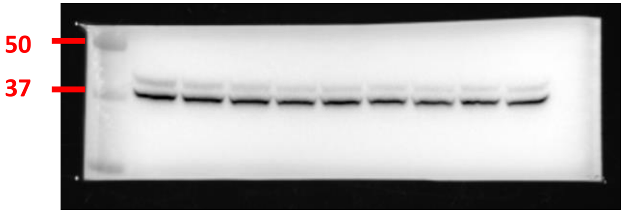

ERK Predicted MW 42-44 Kda

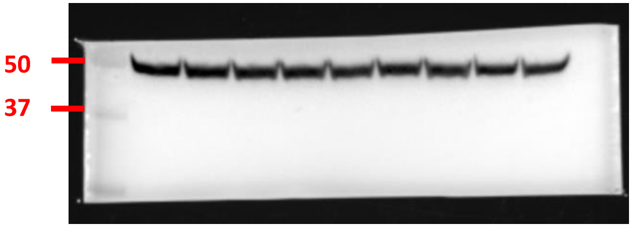

α-tubulin predicted MW 50-55 Kda

antago-miR152

veh ACEA rim

1 2 3 1 2 3 1 2 3 *samples*

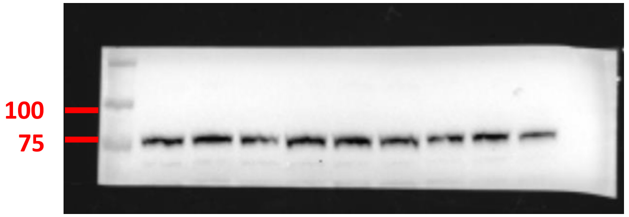

pPKC Predicted MW 77 Kda

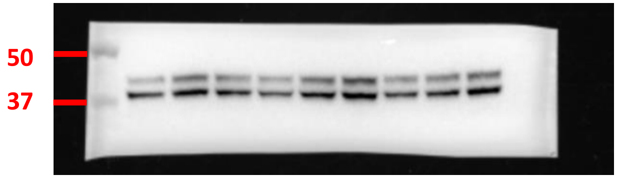

pERK Predicted MW 42-44 Kda

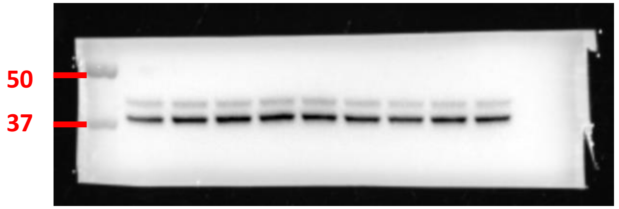

ERK Predicted MW 42-44 Kda

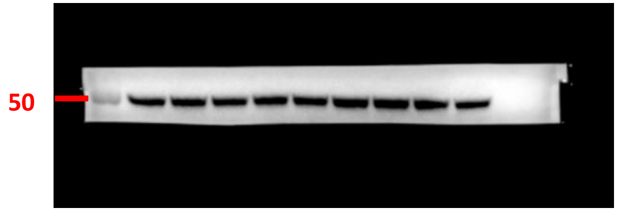

α-tubulin predicted MW 50-55 Kda

Raw blots shown in Supplementary Fig. 6

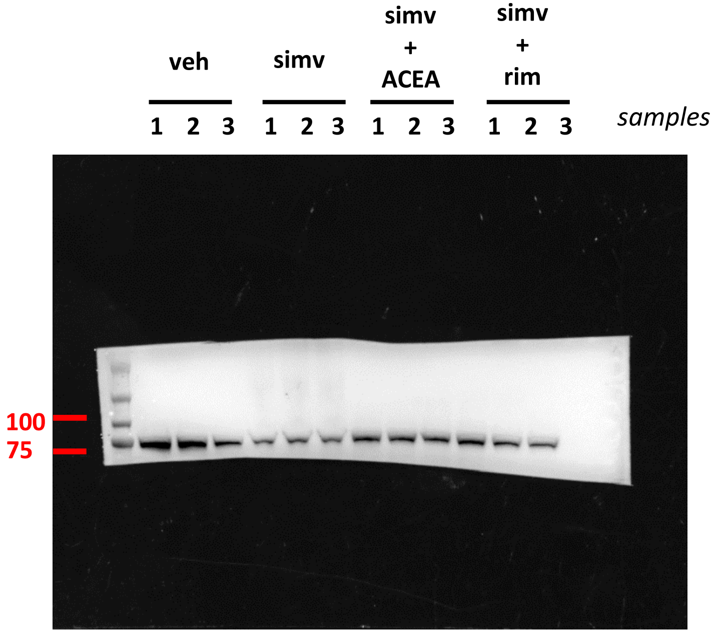

pPKC Predicted MW 77 Kda

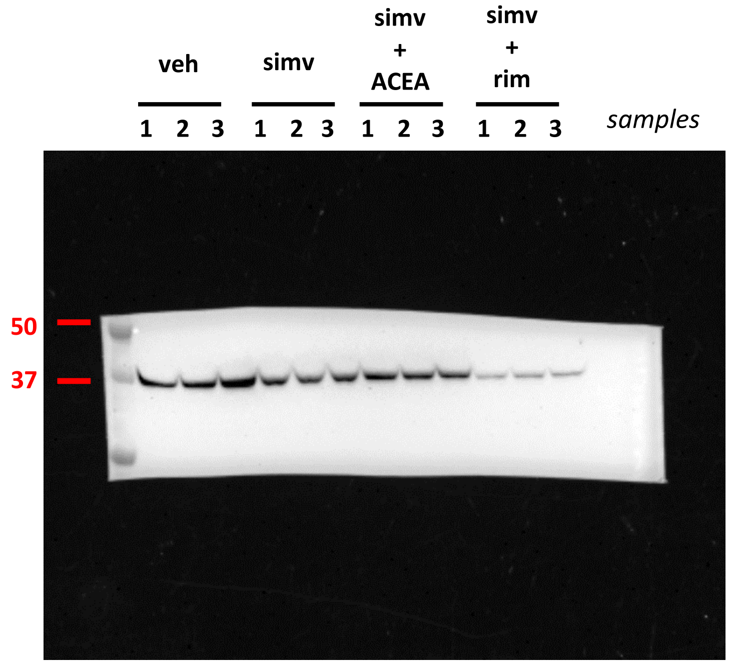

pERK Predicted MW 42-44 Kda

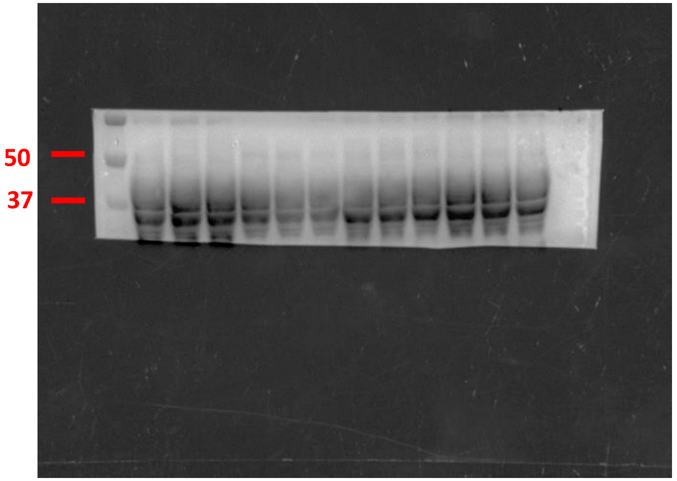

GADPH Predicted MW 36 Kda

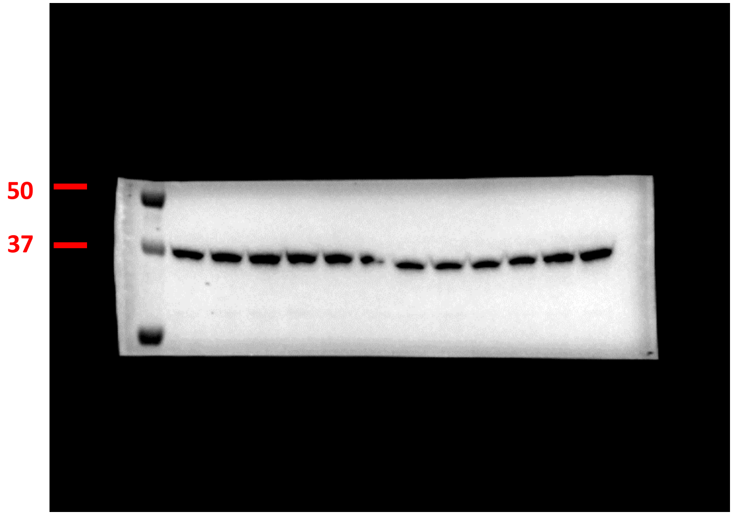

ERK Predicted MW 42-44 Kda
