# Supplementary material for: Dysfunctional endocannabinoid CB1 receptor expression and signaling contribute to skeletal muscle cell toxicity induced by simvastatin
Source: Cell Death Dis. 2023 Aug 23;14(8):544. doi: 10.1038/s41419-023-06080-9 (PMC10447569; doi:10.1038/s41419-023-06080-9)
Supplement: Supplementary file 9 — co-authors agreement [file 41419_2023_6080_MOESM9_ESM.pdf]

# AUTHORS DECLARATION

## Author: Raffaele Capasso

**Re: CDDIS-22-4751RRR Initial Quality Check** Messaggio 3 di 27931

Mittente rafcapas
Destinatario Dr. Sztretye Mónika Tünde
Cc Hilal Kalkan , Vincenzo Di Marzo , e.panza@unina.it , ester.pagano@unina.it , Fabiana Piscitelli , fabio iannotti , Claudia Moriello , giuseppe.ercolano@unina.it
Data Oggi 07:12

Dear all
I confirm my agreement with all the changes that have been made.
Best regards
Raffaele

## Author: Monika Sztretye

**Re: CDDIS-22-4751RRR Initial Quality Check** Messaggio 10 di 27931

Mittente Dr. Sztretye Mónika Tünde
Destinatario Hilal Kalkan
Cc Vincenzo Di Marzo , e.panza@unina.it , ester.pagano@unina.it , Fabiana Piscitelli , fabio iannotti rafcapas@unina.it , Claudia Moriello , giuseppe.ercolano@unina.it
Data Mer 21:13

Dear co-authors,

I confirm my agreement with all the changes that have been made.

Kind regards
Monika Sztretye

## Author: Vincenzo Di Marzo

**RE: CDDIS-22-4751RRR Initial Quality Check** Messaggio 4 di 27927

Mittente vdimarzo
Destinatario 'Fabio Arturo Iannotti' , e.panza@unina.it , ester.pagano@unina.it , fpiscitelli@icb.cnr.it
Cc rafcapas@unina.it , cla_mar97@hotmail.it , hilal.kalkan@criucpq.ulaval.ca , giuseppe.ercolano@unina. sztretye.monika@med.unideb.hu
Data Oggi 04:16

I confirm that I agree with all these changes.
Vincenzo

*Professor Vincenzo Di Marzo, PhD*

**Author: Elisabetta Panza**

## CDDIS-22-4751RRR Initial Quality Check

Messaggio 12 di 27931

Mittente: Ester Pagano
Destinatario: Fabio Arturo Iannotti
Data: Mer 19:27

Dear Fabio,

Many thanks.
I confirm my agreement to all the changes that have been made.
Best regards,
Ester

**Author: Giuseppe Ercolano**

## RE: Fwd: CDDIS-22-4751RRR Initial Quality Check

Messaggio 13 di 27931

Mittente: giuseppe.ercolano
Destinatario: Fabio Arturo Iannotti, Vincenzo Di Marzo, e.panza@unina.it, ester.pagano@unina.it, fpiscitelli@icb.cnr.it
Cc: rafcapas@unina.it, cla_mar97@hotmail.it, hilal.kalkan@criucpq.ulaval.ca, sztretye.monika@med.unideb.hu
Data: Mer 19:25

Dear all,

Hereby, I confirm that I agree with all the changes that have been made.

Best regards
Giuseppe Ercolano

**Author: Elisabetta Panza**

## Re: CDDIS-22-4751RRR Initial Quality Check

Messaggio 14 di 27931

Mittente: Elisabetta Panza
Destinatario: Fabio Arturo Iannotti
Data: Mer 19:14

Dear all,

I confirm that I agree with all the changes made.

Best regards
Elisabetta Panza

**Author: Hilal Kalkan**

## Re: CDDIS-22-4751RRR Initial Quality Check

Messaggio 15 di 27931

Mittente: Hilal Kalkan
Destinatario: Vincenzo Di Marzo, e.panza@unina.it, ester.pagano@unina.it, Fabiana Piscitelli, fabio iannotti
Cc: rafcapas@unina.it, Claudia Moriello, giuseppe.ercolano@unina.it, sztretye.monika@med.unideb.hu
Data: Mer 19:11

winmail.dat (~21 KB)

Dear all,

Hereby, I confirm the changes that have been made.

Kind regards,
Hilal Kalkan,

**Author: Claudia Moriello**

## R: CDDIS-22-4751RRR Initial Quality Check

Messaggio 16 di 27931

Mittente Claudia Moriello

Destinatario Fabio Arturo Iannotti

Data Mer 19:10

Dear Dr Iannotti,
I agree to all the changes made to the text and figures.
Sincerely,
Claudia Moriello

**Author: Fabiana Piscitelli**

## Re: Fwd: CDDIS-22-4751RRR Initial Quality Check

Messaggio 17 di 27931

Mittente Fabiana Piscitelli

Destinatario Fabio Arturo Iannotti

Cc Vincenzo Di Marzo, e.panza@unina.it, ester.pagano@unina.it, rafcapas@unina.it, cla_mar97@hotmail.it, hilal.kalkan@criucpq.ulaval.ca, giuseppe.ercolano@unina.it, sztretye.monika@med.unideb.hu

Data Mer 19:07

Priorità Normale

Dear all,

I confirm that I agree with all these changes.

Best regards
Fabiana Piscitelli
